# CRISPR in MOF Formulation with Enhanced Stability, Activity, and Altered PAM Specificity for Broad‐Spectrum Diagnosis of Bacterial Sepsis

**DOI:** 10.1002/advs.202513439

**Published:** 2025-11-23

**Authors:** Tathagata Pal, Zilong Liu, Meera G. Nair, Juhong Chen

**Affiliations:** ^1^ Department of Bioengineering University of California Riverside CA 92521 USA; ^2^ Division of Biomedical Sciences School of Medicine University of California Riverside CA 92521 USA

**Keywords:** Cas12a variant, CRISPR assay, MOF, PAM‐altered detection, sepsis diagnosis

## Abstract

Sepsis is a life‐threatening condition caused by polymicrobial infections and remains a global health emergency that requires rapid and broad‐spectrum diagnostics. Existing CRISPR‐based assays face two major limitations that restrict their application for sepsis: narrow protospacer adjacent motif (PAM) site compatibility and poor enzyme stability under clinical and environmental stresses. A modular diagnostic platform is presented, CRISPR‐FLEXMO (CRISPR with flexible PAM in metal‐organic framework encapsulation, MOF), which integrates a PAM‐relaxed Cas12a variant (K607R) with a manganese‐coordinated MOF (Mn‐MOF) for stable and specific detection of sepsis‐causing bacteria. The system targets a conserved region upstream of the Shine‐Dalgarno sequence in the 16S rRNA gene containing a universal TTCC PAM, enabling broad‐spectrum detection with a single universal primer pair across Gram‐negative and Gram‐positive pathogens. The K607R variant shows enhanced *cis*‐ and *trans*‐cleavage activity, while Mn‐MOF encapsulation maintains enzyme functionality under ambient, thermal, and chaotropic stress. The assay detects as low as 10 CFU mL^−1^ in bacterial lysates following amplification and achieves 100% sensitivity and specificity in serum samples from 15 sepsis patients and 3 healthy individuals, with no cross‐reactivity to six respiratory viruses. The platform retains over 78% activity after 12 weeks of room‐temperature storage, offering a field‐deployable CRISPR diagnostic solution for next‐generation infectious disease detection.

## Graphical abstract

CRISPR‐FLEXMO (CRISPR with flexible PAM in MOF encapsulation) integrates a PAM‐relaxed Cas12a K607R enzyme with a manganese‐coordinated MOF for broad‐spectrum diagnosis of bacterial sepsis. The MOF enhances activity and preserves functionality under heat, solvent, and chaotropic stress, supporting long‐term room‐temperature storage. The platform delivers robust performance in patient samples of varied etiology and effectively distinguishes bacterial from viral infections.

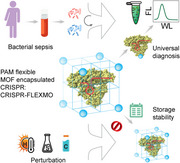

## 1 Introduction

Sepsis remains a major global health threat and is responsible for ≈11 million deaths annually.^[^ ^1^ ^]^ It results from a dysregulated host immune response to infection, which can progress rapidly to systemic inflammation, multiorgan failure, and septic shock.^[^ ^2^ ^]^ The clinical presentation is highly heterogeneous, and the underlying infections are often caused by a wide array of Gram‐negative and Gram‐positive bacteria, including *Escherichia coli*, *Pseudomonas aeruginosa*, *Listeria monocytogenes*, and *Bacillus subtilis*.^[^ ^3^, ^4^ ^]^ A critical determinant of survival is the timely identification of causative pathogens, which enables prompt and targeted therapy.^[^ ^5^ ^]^ However, current diagnostic practices lag behind clinical needs.^[^ ^6^ ^]^ Blood culture, the gold standard, requires several days to deliver results and has poor sensitivity in low bacterial loads.^[^ ^7^ ^]^ Although polymerase chain reaction (PCR)‐based methods offer improved turnaround and specificity, they rely on thermocycling and complex instrumentation, limiting their applicability in point‐of‐care (POC) settings.^[^ ^8^ ^]^ Furthermore, existing molecular diagnostics often target a narrow spectrum of pathogens, overlooking the diversity inherent in clinical sepsis.^[^ ^9^ ^]^ These challenges underscore the urgent need for a rapid, sensitive, and field‐deployable diagnostic platform capable of broad‐spectrum bacterial detection without the requirement of centralized laboratory infrastructure.^[^ ^10^ ^]^

CRISPR (clustered regularly interspaced short palindromic repeats)‐based molecular diagnostics, combining sequence‐specific recognition with collateral signal amplification, offer a promising alternative to conventional methods.^[^ ^11^, ^12^ ^]^ Cas12a enzymes, in particular, have garnered attention due to their ability to *trans*‐cleave single‐stranded DNA reporters upon target recognition,^[^ ^13^ ^]^ enabling fluorescence‐based detection under isothermal conditions.^[^ ^14^ ^]^ This eliminates the need for thermocycling and simplifies assay workflows.^[^ ^15^, ^16^ ^]^ However, the widespread adoption of Cas12a diagnostics has been limited by the enzyme's strict requirement for a specific Protospacer Adjacent Motif (PAM) to recognize target sequences.^[^ ^17^ ^]^ Wild‐type Cas12a primarily recognizes canonical TTTV motifs, reducing its flexibility for broad‐spectrum pathogen detection.^[^ ^18^, ^19^ ^]^ Expanding PAM compatibility through protein engineering is essential to unlock the full potential of Cas12a in diagnostics.^[^ ^20^ ^]^ However, even with enhanced PAM flexibility, effective broad‐spectrum detection requires targeting genomic regions that are highly conserved across diverse bacterial species.^[^ ^21^, ^22^ ^]^ Combining a PAM‐flexible Cas12a variant with a universal sequence could dramatically improve diagnostic inclusivity and reduce false negatives in polymicrobial infections.

Despite the promise of CRISPR diagnostics, a persistent limitation lies in the instability of the CRISPR‐Cas system under ambient and field conditions.^[^ ^23^ ^]^ Enzymatic degradation due to heat, solvents, and proteolytic agents can significantly compromise assay reliability and shelf life.^[^ ^24^ ^]^ To address this limitation, metal‐organic frameworks (MOFs), crystalline materials formed through the coordination of metal ions and organic ligands, offer a compelling solution.^[^ ^25^, ^26^, ^27^, ^28^ ^]^ MOFs provide a confined microenvironment that can protect fragile biomolecules from denaturation and degradation, while permitting substrate access through controlled disintegration.^[^ ^29^, ^30^ ^]^ In particular, squaric acid‐based MOFs are known for their structural tunability, mild degradation profile, and enzyme activity enhancement.^[^ ^31^ ^]^ In addition, metal ions released from MOF nodes can serve as auxiliary cofactors, increasing the catalytic performance of encapsulated enzymes.^[^ ^32^ ^]^ MOF can offer many advantages, including enzyme protection during storage, controlled release under mild conditions, and boosted enzymatic activity, all while being synthetically accessible and compatible with downstream detection readouts. However, the effect of such metal‐node‐based MOFs on the performance of CRISPR‐based diagnosis has not been systematically explored.

Herein, we integrated two novel technologies (protein engineering and enzyme immobilization) to address key limitations in the current CRISPR‐based diagnostics for sepsis. First, we posited that the Cas12a K607R variant, engineered via site‐directed mutagenesis to broaden PAM recognition, would facilitate universal bacterial detection by targeting a conserved TTCC PAM and downstream spacer sequence within the 16S rRNA region. This region lies upstream of the Shine‐Dalgarno sequence and allows amplification with a designed single primer pair conserved across both Gram‐negative and Gram‐positive pathogens. Second, we encapsulated the CRISPR ribonucleoprotein (RNP) complex within a squarate‐based metal‐organic framework, providing structural protection of the CRISPR nuclease and functional enhancement under ambient and clinically relevant stress conditions. Specifically, we designed a comprehensive strategy involving MOF synthesis, enzyme encapsulation, cleavage efficiency benchmarking, and analytical sensitivity assessments using purified DNA, whole‐cell lysates, and isothermal amplification. We further incorporated serum‐based validation with clinical samples and investigated potential off‐target responses using intact viral genomes. Overall, this platform has the potential to advance the field of nucleic acid diagnostics by integrating enzyme engineering with MOF‐based stabilization to support highly robust, portable, and bacteria‐agnostic detection tools for infectious disease management.

## 2 Results and Discussion

### 2.1 Detection Principle and Universal Targeting

**Scheme 1:**
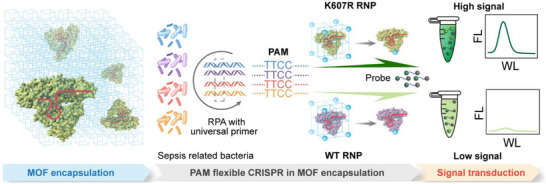
Schematic of the CRISPR‐FLXEMO diagnostic platform integrating the PAM‐flexible Cas12a K607R variant with squaric acid‐based Mn‐MOF encapsulation for stable and broad‐spectrum sepsis diagnostics. Upon Mn‐MOF disassembly, the released Cas12a‐crRNA RNP targets a conserved 16S rRNA region containing a universal TTCC PAM and adjacent spacer. The Cas12a K607R variant exhibits enhanced fluorescence via *trans*‐cleavage, enabling sensitive detection of both Gram‐negative and Gram‐positive bacteria.

Our diagnostic strategy (named CRISPR‐FLXEMO) is built on the integration of a PAM‐flexible CRISPR‐Cas12a system with a squaric acid‐based metal‐organic framework (MOF), designed to achieve stable and broad‐spectrum bacterial detection (**Scheme** **1**). The assay begins with recombinase polymerase amplification (RPA) using a universal primer pair that targets a highly conserved region of the bacterial 16S rRNA gene, located just upstream of the Shine‐Dalgarno sequence. This region includes a universal TTCC protospacer adjacent motif (PAM) and an adjacent spacer sequence, enabling the use of a single primer set across both Gram‐negative and Gram‐positive pathogens. The amplified product serves as the CRISPR target. Upon mild acidic disassembly of the MOF, the encapsulated Cas12a‐crRNA ribonucleoprotein (RNP) complex is released, and the metal nodes (especially manganese, Mn) act as auxiliary cofactors to enhance enzyme activity. Target binding activates the Cas12a *cis*‐cleavage of the amplicons, followed by the *trans*‐cleavage of single‐stranded fluorophore‐quencher (FQ) reporters. This produces a visible green fluorescence signal, indicating the presence of sepsis‐causing bacteria.

To establish a universal CRISPR detection system, we first identified a conserved target site for sepsis‐causing bacteria. Using Clustal Omega and MEGA11, we aligned full‐length sequences within the 16S rRNA gene from representative Gram‐negative and Gram‐positive bacteria, revealing a highly conserved region upstream of the Shine‐Dalgarno sequence. The target region contains a TTCC PAM and an adjacent spacer. A universal primer pair was designed to amplify this 371 bp region and experimentally validated across four bacterial species spanning broad Gram taxa (Table S1, Supporting Information), each yielding the expected amplicon (Figure S1, Supporting Information). Direct sequencing of the bacterial isolates confirmed the conservation of the PAM‐spacer motif, supporting its suitability as a pan‐bacterial target (Figure S2, Supporting Information).

### 2.2 K607R Variant Exhibits Enhanced Cleavage Activity Over Wild‐Type Cas12a

The Cas12a K607R variant was selected by two foundational considerations, including (1) the need to recognize the TTCC PAM and adjacent spacer motif located in a highly conserved region of the bacterial 16S rRNA gene, and (2) the use of universal primers upstream of the Shine‐Dalgarno sequence to enable broad detection of both Gram‐negative and Gram‐positive bacteria. The TTCC and adjacent spacer motif, embedded within phylogenetically conserved domains, offer a strategic anchor point for a universal CRISPR‐based diagnostic. However, wild‐type Cas12a lacks optimal affinity for TTCC, necessitating a rationally designed mutation to improve target engagement.^[^ ^33^ ^]^ Structural and biochemical studies have shown that the K607R mutation enhances DNA binding and cleavage efficiency, particularly when paired with non‐canonical PAMs.^[^ ^34^ ^]^ In this context, the Cas12a K607R variant is uniquely positioned to expand the PAM landscape and elevate the universality of bacterial detection assays. The K607R mutation was introduced into the AsCas12a gene via site‐directed mutagenesis. The mutated plasmid construct was confirmed by Sanger sequencing (Figure S3, Supporting Information). The corresponding protein was successfully expressed, purified, and characterized using SDS‐PAGE analysis (Figure S4, Supporting Information). This groundwork enabled the subsequent benchmarking of cleavage activity and diagnostic performance for the engineered Cas12a enzyme.

**Figure 1:**
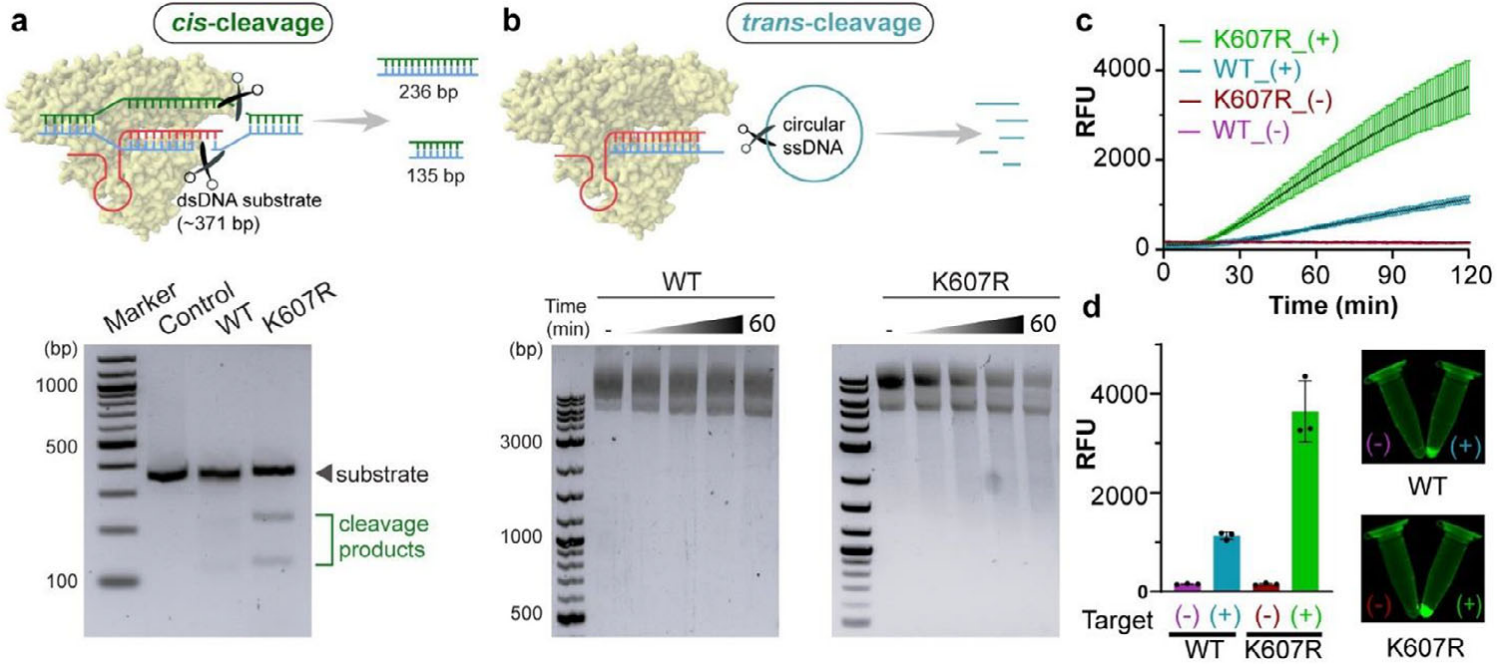
Cas12a K607R variant exhibits enhanced *cis*‐ and *trans*‐cleavage activity compared to wild‐type (WT) Cas12a. a) Schematic of *cis*‐cleavage assay using a 371 bp PCR amplicon generated with universal primers targeting a conserved region upstream of the Shine‐Dalgarno sequence within the 16S rRNA gene. Cleavage at the TTCC PAM site yields 236 bp and 135 bp fragments. Gel electrophoresis confirms superior cleavage efficiency of Cas12a K607R over WT Cas12a, as indicated by more prominent cleavage bands. b) Schematic of *trans*‐cleavage assay, wherein activated Cas12a cleaves a circular single‐stranded DNA (ssDNA) reporter probe upon target recognition. Time‐dependent degradation of ssDNA reveals faster and more extensive cleavage by Cas12a K607R than WT Cas12a. c) Kinetic fluorescence measurements show markedly higher signal output for Cas12a K607R (+) compared to WT Cas12a (+), with minimal background in negative controls. d) Endpoint fluorescence intensities and visual readouts further highlight the robust signal generation enabled by Cas12a K607R, supporting its suitability for diagnostics. Error bars represent the standard deviation obtained in three parallel experiments (*n* = 3).

To validate the superiority of Cas 12a K607R over WT Cas12a, we conducted *cis*‐ and *trans*‐cleavage assays targeting the TTCC PAM site within the conserved 16S rRNA region with designed crRNA (Table S2, Supporting Information). The *cis*‐cleavage efficiency was assessed using a 371 bp double‐stranded DNA (dsDNA) amplicon from *E. coli*, where successful cleavage resulted in fragment sizes of 236 bp and 135 bp (**Figure** **1**a). Gel electrophoresis revealed that Cas12a K607R exhibited significantly stronger cleavage activity than WT, leading to more pronounced cleavage bands. Beyond the *cis*‐cleavage, we investigated the *trans*‐cleavage activity of the system, which governs collateral signal amplification in CRISPR diagnostics. A circular single‐stranded DNA (ssDNA) M13 plasmid was used to assess *trans*‐cleavage through gel electrophoresis, where Cas12a K607R showed near‐complete substrate degradation compared to partial cleavage by the wild‐type (WT) enzyme (Figure 1b). This superior performance aligns with previous findings,^[^ ^35^ ^]^ demonstrating that engineered Cas12a variants with expanded PAM compatibility show higher catalytic efficiency and specificity.

To further confirm this enhancement, we developed a complementary real‐time fluorescence assay using single‐stranded DNA Fluorophore‐Quencher (ssDNA‐FQ) probes. As shown in Figure 1c, the kinetics revealed that Cas12a K607R generated a robust fluorescence signal (≈4000 RFU) in the presence of target dsDNA, nearly four times higher than that of WT Cas12a (≈1000 RFU). This increase in collateral activity is likely attributed to the mutation's effect on target affinity and catalytic efficiency. Fluorescent endpoint analysis further confirmed Cas12a K607R's superior signal generation, as shown by both tube fluorescence images and the corresponding bar graph quantification (Figure 1d). The stark contrast in signal intensity between positive and negative samples underscores the practical advantage of Cas12a K607R in achieving high sensitivity within amplification‐assisted diagnostics. These results confirm that the Cas12a K607R variant significantly outperforms wild‐type Cas12a in both *cis*‐ and *trans*‐cleavage efficiency. The use of a universal primer pair targeting a TTCC PAM‐containing conserved region enables broad‐spectrum bacterial detection across phylogenetically diverse species. By overcoming PAM specificity constraints, the Cas12a K607R variant provides a robust enzymatic foundation for the CRISPR‐FLEXMO assay.

### 2.3 Screening and Characterization of MOFs for CRISPR Encapsulation

MOFs provide a protective environment that stabilizes enzymes and enhances enzymatic activities by modulating the local biochemical environment.^[^ ^31^ ^]^ To enhance the stability and efficiency of CRISPR‐based detection, we screened MOFs made from different metal ions for their suitability as encapsulation matrices. Sodium squarate, the organic linker, was first synthesized in‐house (Figure S5, Supporting Information). Twelve distinct MOFs were subsequently prepared using six different metal nodes–manganese (Mn), magnesium (Mg), calcium (Ca), copper (Cu), iron (Fe), and zinc (Zn)–both without and with RNP encapsulation. Variations in color and texture were observed across the synthesized MOFs, reflecting differences in metal coordination and crystal morphology (Figure S6, Supporting Information).

**Figure 2:**
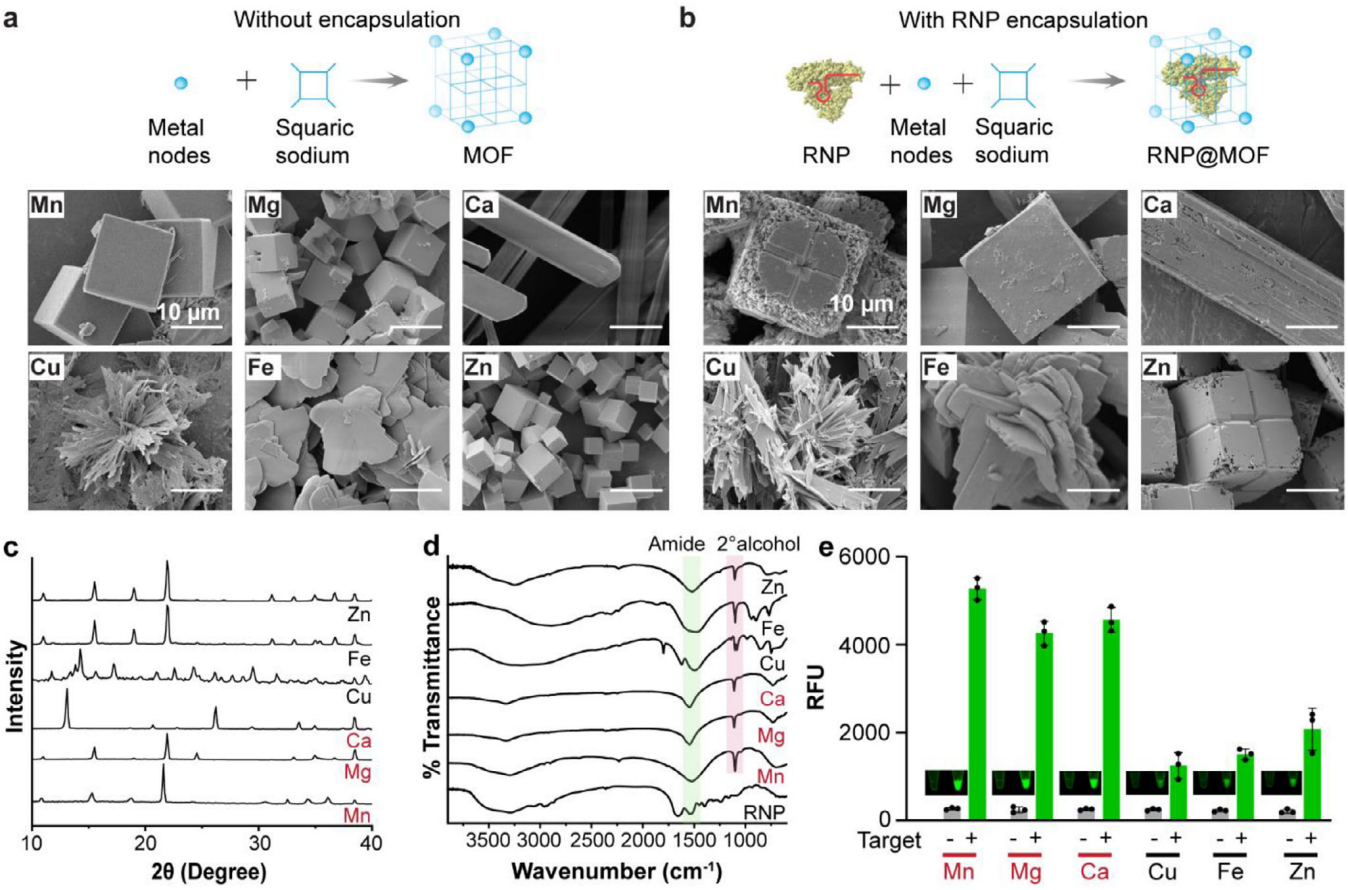
Morphological, structural, and functional characterization of metal‐organic frameworks (MOFs) synthesized with various metal nodes and loaded with Cas12a K607R RNP. Scanning electron microscopy (SEM) images showing the morphological comparison of squarate‐based metal organic frameworks (MOFs) synthesized using six different metal ions, without (panel a) and with (panel b) Cas12a K607R‐crRNA ribonucleoprotein (RNP) encapsulation. Metal ions used: Mn, Mg, Ca, Fe, Cu, and Zn. c) X‐ray diffraction (XRD) patterns demonstrating preserved crystallinity of the MOFs across different metal compositions. d) FTIR spectra confirming successful RNP encapsulation across MOFs. e) Fluorescence signal (RFU) upon MOF disintegration and CRISPR activation, indicating Mn‐MOF exhibits the highest *trans*‐cleavage activity, followed by Mg‐ and Ca‐based MOFs. Cu‐, Fe‐, and Zn‐MOFs yielded significantly lower signal output. Inset: fluorescence tube images showing endpoint signal for each condition. Error bars represent the standard deviation obtained in three parallel experiments (*n* = 3).

The MOFs were characterized using a scanning electron microscope (SEM). As shown in **Figures** **2**a,b and S7 (Supporting Information), the use of different metal ions as anchoring nodes resulted in MOFs with distinct morphologies, which can be attributed to the metal‐specific coordination interactions with the squaric sodium ligand.^[^ ^36^ ^]^ The MOFs using Mn, Mg, and Zn exhibited cubic structures, driven by their octahedral (Oh) coordination geometry, allowing isotropic growth and a well‐defined cubic framework. The one with Ca, having a larger ionic radius, led to rectangular rod‐like structures, reflecting its tendency for higher coordination numbers (7–9 ligands) and anisotropic crystal growth. Cu‐MOFs adopted needle‐ or sheet‐like morphologies, influenced by Jahn‐Teller distortion, which elongates bonds in one direction and favors 2D layered growth. Fe MOFs exhibited petal‐ or flower‐like formations, resulting from redox heterogeneity that caused disordered nucleation and defective crystal growth.^[^ ^37^ ^]^

The crystallinity of the synthesized MOFs was assessed using X‐ray diffraction (XRD) analysis (Figure 2c for the MOF with RNP encapsulation and Figure S8 (Supporting Information) for the MOF without any encapsulation). We confirmed phase purity and structural stability, which is consistent with prior reports on squaric sodium‐based MOFs.^[^ ^31^ ^]^ The Mn‐based MOF exhibited a distinct [101] cubic plane with peaks similar to the cited reports.^[^ ^31^ ^]^ Notably, the crystal patterns were not disturbed after encapsulation of RNP. Fourier‐transform infrared spectroscopy (FTIR) was then used to analyze the incorporation of CRISPR RNPs (Figure 2d). The amide and secondary alcohol peaks in the spectra are aligned with previous studies on enzyme‐functionalized MOFs.^[^ ^38^ ^]^

To determine the effect of MOF composition on sensing signal, we evaluated CRISPR‐based fluorescence readouts upon MOF disintegration. As shown in Figure 2e, Mn‐MOF exhibited the highest fluorescence signal, outperforming other metal‐based MOFs. While Mg is typically the preferred cofactor for Cas12a, the NEB 2.1 reaction buffer already contains Mg, allowing Mn to function as a cofactor and further enhancing enzyme activity. The superior performance of Mn‐MOF is consistent with recent findings showing that divalent Mn ions enhance Cas12a cleavage with Mg ions. This enhancement is attributed to the stabilization of the enzyme's active site, which has been reported to facilitate improved *trans*‐cleavage kinetics in CRISPR‐based detection assays.^[^ ^32^ ^]^ The average particle size of encapsulated Mn‐MOFs was 19.95 ± 1.86 µm (Figure S9, Supporting Information), with an apparent zeta potential of −8.9 ± 1.6 mV (Figure S10, Supporting Information). The encapsulation efficiency, determined from BCA analysis, was 88.73% (Figure S11, Supporting Information). Given its superior crystallinity and ability to enhance CRISPR‐Cas activity, Mn‐MOF was selected as the optimal candidate for further optimization of the MOF‐CRISPR assay.

### 2.4 Optimization of the MOF‐CRISPR Assay

**Figure 3:**
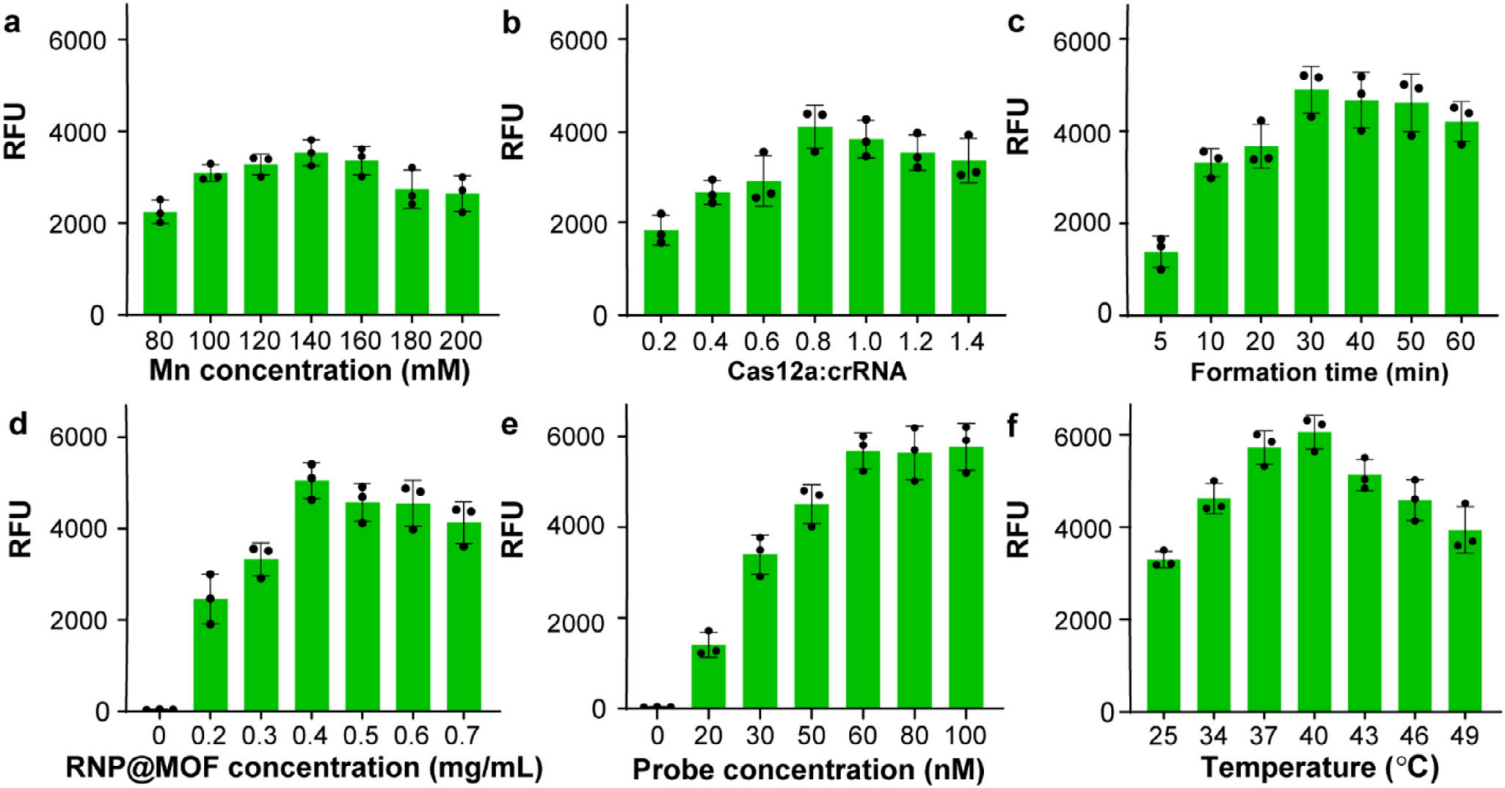
Optimization of key parameters for the CRISPR‐FLEXMO detection system. a) Effect of Mn^2^⁺ concentration during MOF synthesis on fluorescence output. b) Optimization of Cas12a: crRNA molar ratio. c) Influence of MOF formation time on RNP functionality. d) Signal output as a function of RNP@MOF concentration. e) Effect of probe concentration on *trans*‐cleavage signal. f) Reaction temperature screening. Error bars represent the standard deviation obtained in three parallel experiments (*n* = 3).

Before optimizing the detection assay, we validated the system design using component fluorescence analysis. As shown in Figures S12 and S13 (Supporting Information), negligible background fluorescence without target DNA was observed, and the encapsulated Cas12a K607R variant demonstrated significantly enhanced *trans*‐cleavage activity compared to wild‐type Cas12a. To maximize the detection efficiency of the CRISPR‐FLEXMO system, we systematically optimized key reaction parameters, including metal ion concentration, Cas12a‐to‐crRNA ratio, MOF formation time, RNP loading, probe concentration, and reaction temperature (**Figure** **3**). Each parameter was varied independently to assess its impact on fluorescence signal intensity, ensuring the highest possible sensitivity while maintaining assay robustness.

As illustrated in Figure 3a, fluorescence intensity increased with Mn^2^⁺ concentration, peaking at 140 mm. Lower concentrations likely resulted in incomplete MOF formation and insufficient enzyme encapsulation. Conversely, excessive Mn^2^⁺ could disrupt coordination geometry, potentially impeding effective Cas12a release. This trend is consistent with previous findings that high levels of Mn^2^⁺ can interfere with nucleic acid binding through non‐specific electrostatic shielding effects.^[^ ^32^ ^]^ Figure 3b shows that increasing the Cas12a‐to‐crRNA ratio up to 0.8 improved RNP complex formation and signal output. Beyond this point, no further gain was observed, likely due to unbound Cas12a introducing competitive inhibition. Similarly, a 30‐min MOF formation time produced the highest signal (Figure 3c), suggesting this duration provides optimal crystallinity for stable encapsulation while allowing efficient enzyme release. Shorter or longer durations either compromised the crystal structure or reduced the release efficiency.

As shown in Figure 3d, increasing RNP@MOF concentration up to 0.4 mg mL^−1^ enhanced fluorescence, reflecting increased CRISPR activity. However, concentrations above 0.4 mg mL^−1^ did not further improve the signal, suggesting saturation of available target sequences or possible quenching effects due to high enzyme density. These observations align with previous reports where excessive RNP concentration led to aggregation and steric hindrance, ultimately impairing cleavage efficiency. Reporter probe concentration also influenced detection range, with signal intensity rising up to 60 nm and plateauing thereafter (Figure 3e), consistent with classical enzyme kinetics where excess substrate no longer enhances activity. Temperature optimization showed that Cas12a activity peaked at 40 °C, while higher temperatures caused signal decline, likely due to enzyme denaturation (Figure 3f). These results collectively define the optimal conditions to balance enzyme stability, MOF integrity, and detection sensitivity.^[^ ^39^ ^]^

Furthermore, to evaluate the impact of free and framework‐associated Mn^2+^ on RNP activity, a comparative study was performed using varying Mn^2+^ concentrations (0–20 mm) and Mn‐MOF formulations. The fluorescence response exhibited a distinct concentration‐dependent profile, where moderate Mn^2+^ levels (≤1 mm) enhanced Cas12a activity, whereas higher concentrations (≥5 mm) caused a marked reduction in signal intensity, likely due to RNP denaturation. (Figure S14, Supporting Information) Consistently, the Mn‐MOF encapsulated RNP exhibited a stronger and more sustained fluorescence response than the equivalent Mn^2+^‐assisted assay, indicating that the Mn^2+^ ions released gradually from the MOF framework contributed to enzymatic activation without inducing denaturation (Figure S15, Supporting Information).^[^ ^31^ ^]^ These results highlight the dual role of Mn^2+^ in both framework stabilization and catalytic enhancement, confirming that controlled release from the MOF microenvironment preserves RNP functionality under optimal assay conditions.

Through systematic optimization, we identified 140 mm Mn^2^⁺ for synthesis, a Cas12a‐to‐crRNA ratio of 0.8, a 30 min MOF formation time, 0.4 mg mL^−1^ RNP@MOF concentration, 60 nm probe concentration, and a reaction temperature of 40 °C as the ideal conditions for maximizing detection sensitivity. These parameters will be applied for further sepsis diagnostics, ensuring a fine balance between enzyme stability, MOF integrity, and signal amplification.

### 2.5 Performance Evaluation of the CRISPR‐FLEXMO Assay

**Figure 4:**
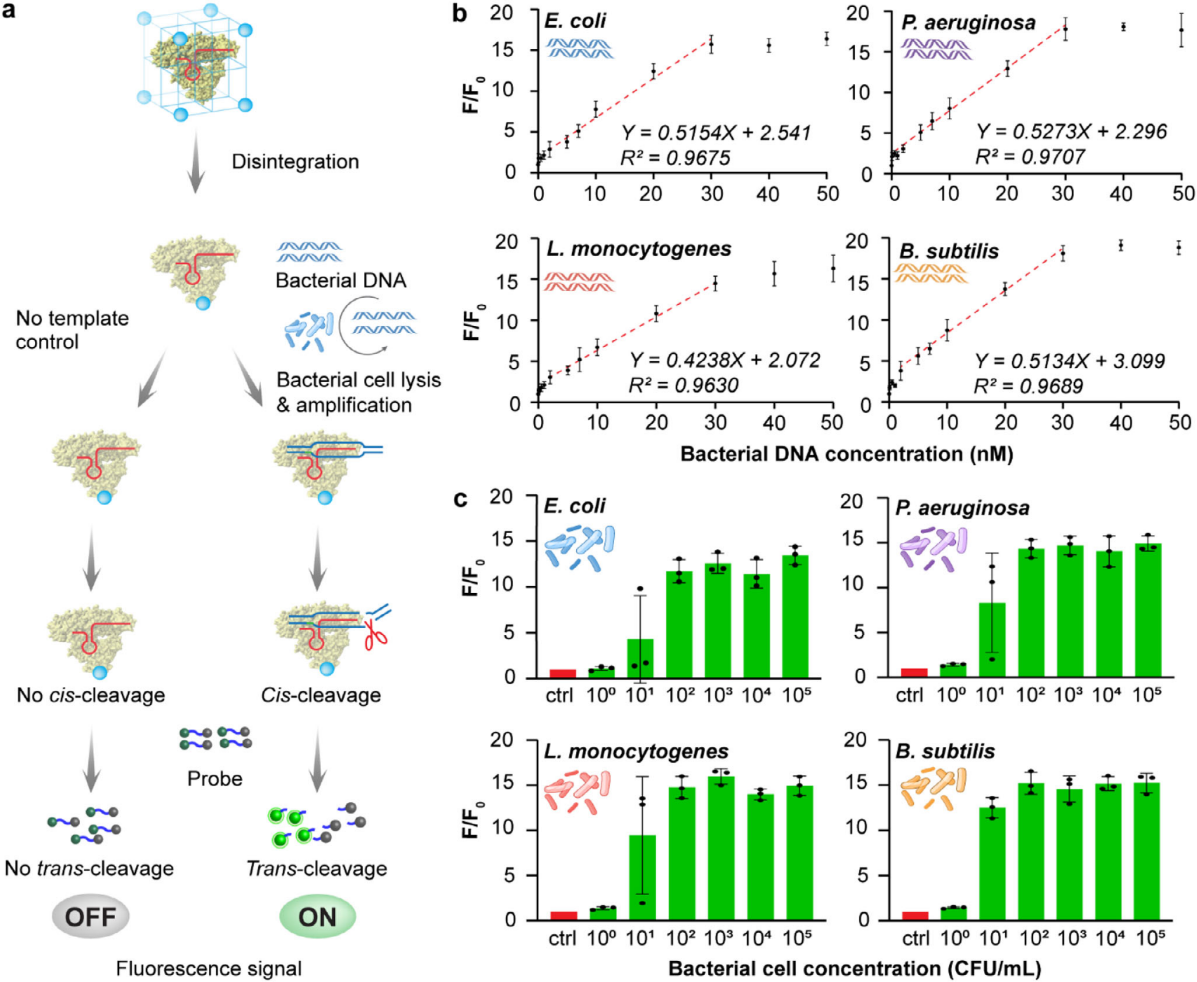
Analytical performance of the CRISPR FLEXMO system for detecting Gram‐negative and Gram‐positive sepsis‐related bacteria using PCR amplicons and whole‐cell lysates. a) Schematic illustration of the detection workflow using the CRISPR FLEXMO system. b) Fluorescence response (F/F_0_) to increasing concentrations of PCR‐amplified bacterial DNA: Gram‐negative *Escherichia coli* and *Pseudomonas aeruginosa*; Gram‐positive *Listeria monocytogenes* and *Bacillus subtilis*. Strong linear correlations (R^2^ > 0.96) were observed within the 1–30 nm range across all species. c) Whole‐cell detection using serial dilutions (10^0^–10^5^ CFU mL^−1^), followed by PCR amplification and CRISPR activation. The platform enables reliable detection down to 10^1^ CFU mL^−1^ for both Gram‐negative and Gram‐positive bacteria. F/F_0_ represents the normalized fluorescence signal, where F is the measured fluorescence intensity at each condition, and F_0_ is the baseline fluorescence in the absence of target DNA. Error bars represent the standard deviation obtained in three parallel experiments (*n* = 3).

Next, we evaluate the overall performance of the optimized CRISPR‐FLEXMO assay (**Figure** **4**a) and assess its analytical sensitivity, specificity, and applicability for detecting sepsis‐causing pathogens. The detection limit (LOD) was determined using serial dilutions of genomic DNA from representative Gram‐negative and Gram‐positive bacterial species: *E. coli*, *P. aeruginosa*, *L. monocytogenes*, and *B. subtilis*. The fluorescence signal (F/F_0_) was plotted against DNA concentration, revealing a robust linear correlation across the tested range (Figure 4b). Notably, the system exhibited a dynamic detection range spanning nanomolar (nM) DNA concentrations, with a clear signal saturation at higher inputs. The LOD for each bacterium was derived from the standard deviation of the negative control (S_y_) and the slope (S) of the regression curve, calculated as LOD = 3.3 × (S_y_/S).^[^ ^39^ ^]^ The results, summarized in Table S3 (Supporting Information), indicate exceptional sensitivity for bacterial DNA detection, with LOD values ranging from 5.92 to 6.67 nm. And *P. aeruginosa* exhibited the lowest LOD (5.92 nm), while *L. monocytogenes* had a slightly higher threshold (6.67 nm).

For whole bacterial detection, cultures were serially diluted from 10^0^ to 10^5^ CFU mL^−1^ and lysed to release genomic DNA for amplification. The fluorescence response exhibited a dose‐dependent trend, demonstrating a strong correlation between bacterial load and signal intensity (Figure 4c). Notably, the assay could detect bacterial concentrations as low as 10 CFU mL^−1^, underscoring its applicability for early‐stage infection detection. These findings confirm the CRISPR‐FLEXMO assay's high sensitivity and versatility for detecting sepsis‐associated bacteria.

To further validate the universality of the CRISPR‐FLEXMO platform, we extended the assay to an expanded bacterial panel comprising eight clinically relevant pathogens. The fluorescence kinetics demonstrated uniform and strong signal responses for all target‐positive samples (Figure S16a, Supporting Information), confirming consistent assay activation across species. Endpoint analysis further revealed comparable detection efficiency for four Gram‐negative bacteria (*Escherichia coli*, *Pseudomonas aeruginosa*, *Salmonella enterica*, and *Salmonella typhimurium*) and four Gram‐positive ones (*Listeria monocytogenes*, *Bacillus subtilis*, *Staphylococcus aureus*, and *Staphylococcus epidermidis*) (Figure S16b, Supporting Information). Together, these results underscore the assay's reliability and broad‐spectrum applicability across diverse bacterial taxa, demonstrating its potential as a universal diagnostic platform for sepsis‐associated infections.

### 2.6 Stability of the CRISPR‐FLEXMO Complex

**Figure 5:**
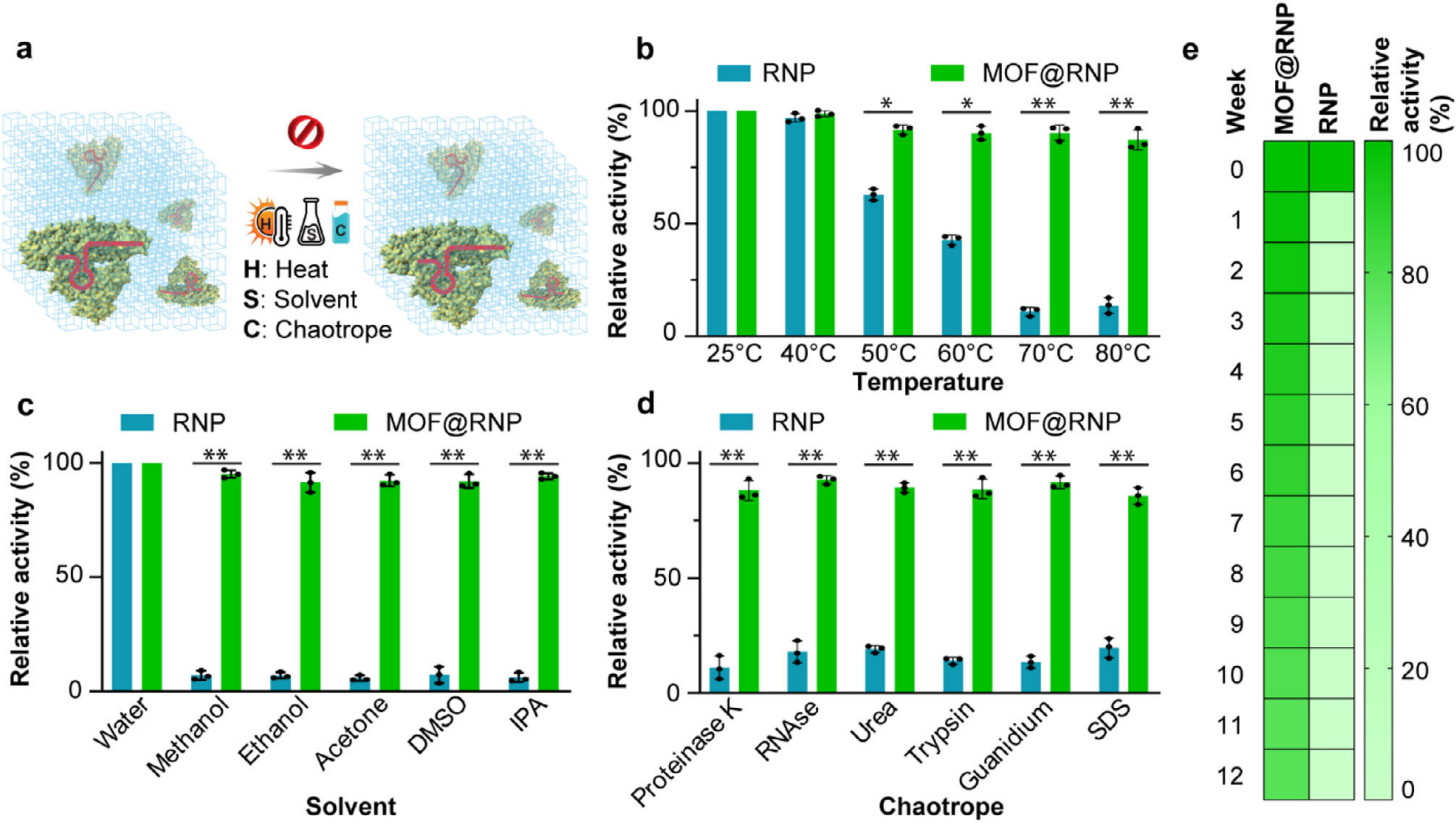
Stability evaluation of MOF‐encapsulated Cas12a K607R CRISPR‐Cas12a (MOF@RNP) under stress conditions and during extended storage. a) Schematic illustrating MOF stability under stress challenges–heat, solvents, and chaotropes, b) Thermal stability: MOF@RNP retains over 80% relative activity up to 80 °C, whereas free RNP loses activity rapidly above 50 °C. c) Solvent tolerance: MOF@RNP remains functional in various organic solvents (methanol, ethanol, acetone, DMSO, IPA), while unencapsulated RNP shows substantial activity loss. d) Resistance to chaotropic and enzymatic agents: MOF@RNP maintains high activity after exposure to Proteinase K, RNase, Urea, Trypsin, Guanidinium, and SDS; free RNP is largely inactivated. e) Long‐term storage stability: MOF@RNP preserves >78% activity over 12 weeks at ambient conditions, compared to continuous degradation observed in unencapsulated RNP. All data expressed as relative fluorescence‐based activity normalized to untreated controls. Student's t‐test shows statistical significance: ^*^ *p* < 0.05 and ^**^ *p* < 0.01. Error bars represent the standard deviation obtained in three parallel experiments (*n* = 3).

The long‐term stability of the CRISPR‐FLEXMO complex is a crucial parameter for real‐world diagnostic applications, ensuring robust performance during storage and transportation. Here, we examined the stability of the CRISPR‐FLEXMO complex under various environmental stressors (**Figure** **5**a), further establishing its suitability for clinical deployment. To determine thermal resilience, the CRISPR‐FLEXMO complex was subjected to increasing temperatures ranging from 50 to 80 °C. The system maintained over 90% of its activity at 50 and 60 °C, while the unprotected CRISPR enzyme (RNP alone) showed rapid degradation. At 80 °C, CRISPR‐FLEXMO retained significant activity (≈80%), whereas the free RNPs were nearly inactivated (Figure 5b). This underscores the protective role of the MOF in preventing heat‐induced enzyme denaturation. Unlike porous MOFs, which often suffer from enzyme leaching and structural degradation, the non‐porous nature of the Mn‐squaric acid‐based MOFs employed in this study provides an enclosed and protective environment for the encapsulated RNP.^[^ ^31^ ^]^ This structural advantage minimizes enzymatic degradation and prevents external environmental stressors from compromising assay performance.

We further assessed the stability of the MOF‐CRISPR system in the presence of common solvents, including methanol, ethanol, and acetone. Unlike free CRISPR enzymes, which exhibited significant activity loss upon solvent exposure, the MOF‐encapsulated system remained highly stable. Notably, fluorescence output remained above 90% in methanol and ethanol and above 85% in acetone (Figure 5c). To simulate extreme denaturing conditions, the CRISPR‐FLEXMO complex was exposed to chaotropic agents, such as guanidium chloride, urea, trypsin, RNase, sodium dodecyl sulfate (SDS), and proteinase K. The fluorescence response of the free RNPs declined sharply under these conditions, reflecting rapid RNP degradation. However, MOF‐encapsulated RNPs retained over 80% of their activity, even in the presence of high concentrations of chaotropic agents (Figure 5d). This remarkable stability suggests that the Mn‐squarate‐MOF provides an enclosed environment that protects CRISPR proteins from proteolytic digestion and chemical denaturation, a key advantage for long‐term storage and point‐of‐care deployment. These results suggest that the MOF structure effectively shields the enzyme from solvent‐induced and chemical denaturation, supporting its use in diverse sample processing environments and enabling long‐term storage and point‐of‐care deployment.

To evaluate stability, the CRISPR‐FLEXMO complex was stored at room temperature and periodically tested over 12 weeks. Remarkably, the fluorescence signal intensity remained consistent throughout the study period, with around 78% activity retention (Figure 5e). These results indicate that the Mn‐MOF effectively preserves CRISPR activity for extended durations, offering a practical solution for point‐of‐care (POC) diagnostics where prolonged storage stability is essential. The exceptional stability of the MOF‐CRISPR complex can be attributed to the robust structural integrity of the Mn‐MOF scaffold, as confirmed by X‐ray diffraction (XRD) analysis showing conserved crystallinity even after exposure to thermal, solvent, and proteolytic stress (Figure S17, Supporting Information). The assay retained its functionality when exposed to temperature variations, simulating potential fluctuations during transportation and on‐site deployment. The robustness of the MOF‐CRISPR system underscores its suitability for field applications, particularly in resource‐limited settings where cold‐chain storage may be impractical.^[^ ^40^ ^]^ A comparative summary of conventional protein preservation methods and the CRISPR‐FLEXMO approach is presented in Table S4 (Supporting Information). The combination of enhanced stability, prolonged storability, and protection against external stressors positions the MOF‐CRISPR system as a highly reliable diagnostic tool for bacterial detection.

### 2.7 Translational Validation of the Diagnostic Platform in Clinical Samples

**Figure 6:**
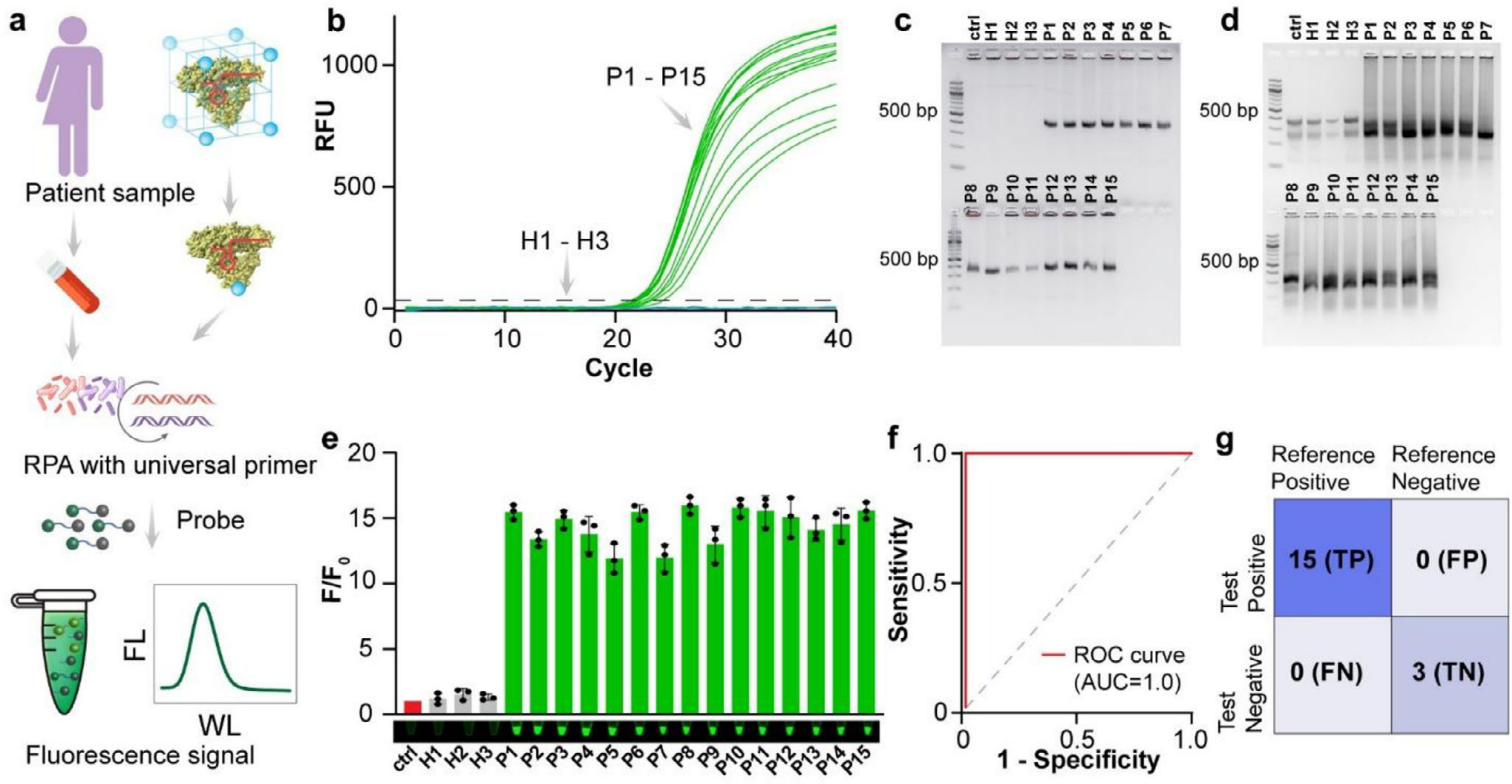
Clinical validation of the MOF‐encapsulated Cas12a K607R CRISPR‐Cas12a system for detection of bacterial DNA in patient serum samples. a) Schematic overview of the diagnostic workflow: patient sample collection, MOF disintegration, isothermal amplification using RPA with universal primers targeting the 16S rRNA gene, followed by CRISPR activation and fluorescence‐based detection. b) Quantitative PCR (qPCR) amplification curves for 15 patient samples (P1–P15) and 3 healthy controls (H1–H3), ctrl: purchased human serum as no target control. c) Endpoint PCR product of the clinical samples, showing strong bands for patient‐derived DNA. d) RPA products used for CRISPR detection, demonstrating compatibility and consistent amplification across patient samples. e) Fluorescence signal (F/F_0_) output of the MOF‐CRISPR assay confirms positive detection in all patient samples and negligible background in negative controls (Fluorescence image of the reaction product in the inset). f) ROC analysis indicating high diagnostic performance. g) Confusion matrix comparing CRISPR results with reference diagnosis (qPCR): 15 true positives (TP), 3 true negatives (TN), and no false positives (FP) or false negatives (FN), demonstrating 100% sensitivity and specificity. F/F_0_ represents the normalized fluorescence signal, where F is the measured fluorescence intensity at each condition, and F_0_ is the baseline fluorescence in the absence of target DNA. Error bars represent the standard deviation obtained in three parallel experiments (*n* = 3).

Lastly, we assess the detection performance of our developed CRISPR‐FLEXMO for sepsis diagnosis in complex biological matrices, specifically patient serum samples, to confirm its translational potential. A cohort of fifteen patients (P1–P15) with clinically suspected sepsis of diverse etiology (Table S5, Supporting Information), three healthy individuals (H1–H3), and one no‐target serum control were enrolled for blinded diagnostic testing. As shown in **Figure** **6**a, the overall detection workflow illustrates the sequential process, from patient sample collection and recombinase polymerase amplification (RPA) with universal primers targeting the conserved 16S rRNA region, to subsequent fluorescence‐based CRISPR detection using the CRISPR‐FLEXMO system.

SYBR Green Quantitative PCR (qPCR) was employed as a clinical benchmark. Robust amplification curves were observed exclusively in patient samples, while all healthy controls and no target control remained minimal (Figure 6b). These results confirmed the presence of bacterial DNA and provided a reference standard for evaluating the CRISPR‐FLEXMO assay. To further validate target‐specific amplification, we performed conventional PCR and RPA on all clinical samples. The resulting amplicons from conventional PCR showed consistent 371 bp bands across patient samples, with no detectable bands in the controls (Figure 6c). These findings confirm the fidelity of the universal primer set and its suitability for broad bacterial detection. As expected, RPA products did not yield consistently sharp 371 bp bands across patient samples (Figure 6d). This is likely due to the unpurified nature of the RPA reaction and the longer amplicon size required by the universal primer set.^[^ ^41^ ^]^ Unlike PCR, RPA often generates diffuse or smeared bands in gel electrophoresis because of its continuous strand displacement mechanism, lower primer‐binding stringency, and the presence of non‐specific by‐products, especially in crude clinical matrices such as serum. Even the healthy controls (H1–H3) showed bands near the expected size, which may reflect partial amplification or non‐specific priming. These observations highlight that RPA, along with universal primers, does not provide a reliable visual indicator of successful healthy and sepsis case differentiation.

In contrast, when the same RPA products were processed through the CRISPR‐FLEXMO detection system, a clear functional distinction was observed. All patient samples produced strong target‐specific fluorescence signals (F/F_0_ > 15), while the healthy controls and no‐template controls showed negligible background (Figure 6e). This confirms that the TTCC PAM site and downstream target region were successfully amplified and recognized. The CRISPR‐based readout, therefore, serves as a more definitive and reliable indicator of amplification success than gel electrophoresis. In this workflow, RPA serves as a rapid amplification tool, but it is the CRISPR‐FLEXMO system that provides the diagnostic precision needed for accurate detection. Diagnostic accuracy was quantified using receiver operating characteristic (ROC) analysis. The area under the curve (AUC) was 1.0, indicating complete classification accuracy (Figure 6f). Confusion matrix analysis confirmed 100% sensitivity and 100% specificity, with no false positives or false negatives detected (Figure 6g). These results mark a significant advancement in the field of CRISPR diagnostics by demonstrating robust, accurate, and amplification‐compatible detection of polymicrobial infections directly from clinical specimens.

To rigorously assess off‐target tolerance and distinguish bacterial from viral infections, we evaluated the specificity of the CRISPR‐FLEXMO assay against genomic material from six common respiratory viruses: H1N1, H7N1, Influenza B, MERS‐CoV, SARS‐CoV‐2, and HCoV‐NL63 (Figure S18, Supporting Information). These viral genomes were subjected to the same RPA protocol used for bacterial targets. As shown in Figure S18 (Supporting Information), none of the viral samples yielded detectable fluorescence signals, while the groups after mixing *E. coli* exhibited strong amplification. This confirms that the system selectively detects bacterial DNA and does not cross‐react with viral nucleic acids, thereby supporting its potential to differentiate bacterial sepsis from viral syndromes. These findings further reinforce the assay's clinical relevance and diagnostic specificity. A comparative summary of the CRISPR‐FLEXMO platform with existing point‐of‐care diagnostic technologies, including LAMP‐CRISPR and rapid immunoassays, is provided in Table S6 (Supporting Information) to contextualize its analytical performance, clinical applicability.

Taken together, the ability of the CRISPR‐FLEXMO platform to distinguish infected individuals from healthy controls with high precision, without the need for complex instrumentation, makes it a promising candidate for decentralized sepsis diagnostics. The robust detection in crude serum samples and the system's specificity against interfering backgrounds highlight its suitability for real‐world clinical settings. By combining enzyme engineering with MOF‐enabled protection and broad‐spectrum targeting, this platform overcomes key barriers to field deployment. It offers enhanced stability under ambient conditions, functional integrity during long‐term storage, and diagnostic reliability in resource‐limited environments. These features establish a foundation for next‐generation nucleic acid diagnostics with practical translational potential.

## 3 Conclusion

In this study, we developed a MOF‐encapsulated CRISPR‐Cas12a platform (CRISPR‐FLEXMO) that enables stable, sensitive, and broad‐spectrum detection of sepsis‐causing bacteria. By integrating a PAM‐relaxed Cas12a K670R variant with a Mn‐squarate‐based MOF matrix, we achieved sequence recognition at a conserved TTCC PAM site and its adjacent spacer region within the 16S rRNA gene, using universal primers compatible with both Gram‐positive and Gram‐negative bacteria. The MOF encapsulation conferred substantial protection to the CRISPR system, retaining over 78% enzymatic activity after 12 weeks of ambient storage and resisting degradation from heat, solvents, and chaotropic agents. The assay reliably distinguished infected patient serum with 100% sensitivity and specificity, and showed no cross‐reactivity with diverse respiratory viruses.

These results establish a modular and isothermal amplification‐compatible platform that brings together enzyme engineering and materials science to enable field‐ready sepsis diagnostics. The ability to function without a cold chain and remain bacteria‐specific without false positives marks a significant advance over conventional methods when detecting broad‐spectrum infections in complex clinical matrices. Looking forward, this strategy offers a promising foundation for expanding CRISPR diagnostics toward universal viral detection and species‐level bacterial differentiation. Future work will focus on adapting the system to other conserved genomic regions and leveraging CRISPR's programmability for multi‐pathogen panels, further advancing precision diagnostics for global health applications.

## 4 Experimental Section

### Materials and Reagents

Squaric acid, sodium oxide, manganese chloride tetrahydrate (MnCl_2_·4H_2_O), magnesium chloride hexahydrate (MgCl_2_·6H_2_O), calcium chloride dihydrate (CaCl_2_·2H_2_O), copper(II) chloride dihydrate (CuCl_2_·2H_2_O), ferric chloride hexahydrate (FeCl_3_·6H_2_O), and zinc nitrate hexahydrate (Zn(NO_3_)_2_·6H_2_O) were purchased from Sigma–Aldrich (St. Louis, MO, USA). MES hydrate (2‐(N‐morpholino) ethanesulfonic acid), used for MOF disassembly, was also obtained from Sigma–Aldrich. Ethanol and 10% aqueous ethanol were prepared using absolute ethanol (ThermoFisher Scientific). Q5 High‐Fidelity PCR Master Mix and M13 single‐stranded DNA (ssDNA) were purchased from New England Biolabs (Ipswich, MA, USA). Recombinase polymerase amplification (RPA) reagents were obtained from TwistDx (Maidenhead, UK). Synthetic bacterial 16S rRNA gene fragments were ordered from Twist Bioscience (San Francisco, CA). CRISPR RNA (crRNA) sequences were synthesized by Integrated DNA Technologies (IDT, Coralville, IA, USA). All fluorescence measurements were performed using an Agilent BioTek Synergy H5 Hybrid Multi‐Mode Reader (Fisher Scientific, Waltham, MA, USA). The WT Cas12a and engineered Cas12a K607R used in this study were expressed and purified as described in the following sections.

### Identification of Conserved 16S rRNA Region and Design of Universal Primers

To identify a conserved region suitable for universal bacterial detection, full‐length 16S rRNA gene sequences from representative Gram‐negative (*Escherichia coli*, *Pseudomonas aeruginosa*) and Gram‐positive (*Listeria monocytogenes*, *Bacillus subtilis*) species were retrieved from the NCBI GenBank database. Multiple sequence alignment was performed using Clustal Omega (EMBL‐EBI), and conserved domains were visualized in MEGA11 software. A highly conserved segment upstream of the Shine‐Dalgarno sequence, containing a TTCC protospacer adjacent motif (PAM) and an adjacent downstream spacer, was identified as the CRISPR‐Cas12a target site.

Universal primer pairs flanking this region were designed using Primer3Plus with optimal GC content, melting temperature (Tm), and product length. The resulting primer pair yielded a 371 bp amplicon. In silico validation was performed using NCBI Primer‐BLAST against the RefSeq Representative Genome Database (organism restricted to Bacteria), confirming broad coverage across phylogenetically diverse strains. For further experimental validation, sequencing data from four clinical bacterial isolates using the reverse primer (*E. coli*, *P. aeruginosa*, *L. monocytogenes*, and *B. subtilis*) were aligned using T‐Coffee. The alignment confirmed the presence and conservation of the TTCC PAM + spacer target region across species. Primers were synthesized by Integrated DNA Technologies (IDT, Coralville, IA) and used in both qPCR benchmarking and RPA‐assisted amplification for CRISPR‐based detection.

### Site‐Directed Mutagenesis of Cas12a K607R Variant

The single‐point mutation (K607R) in the AsCas12a gene was introduced using site‐directed mutagenesis with Vazyme site‐directed mutagenesis software (Vazyme Biotech Co., Ltd. The codon for lysine (AAA) at position 607 was mutated to arginine (CGC). The forward amplification primer included the target substitution and was designed with a homologous sequence length of 17 bp, a melting temperature (Tm) of 60.9 °C, and a GC content of 52.9%. The sequence was as follows: Forward primer: AATGATCCCGcgcTGTTCCACACAGCTGAAAGCC. The reverse amplification primer, designed to flank the same region, had a Tm of 74.3 °C: AACAgcgCGGGATCATTTTTGCTGCATCCGGA. PCR amplification and DpnI digestion were performed according to the manufacturer's protocol for seamless site‐directed mutagenesis. The resulting plasmid was transformed into competent *E. coli* DH5α cells for propagation and sequence verification.

### Expression and Purification of WT Cas12a and Cas12a K607R Variant

Following plasmid verification, both the wild‐type *Lachnospiraceae* bacterium Cas12a (WT Cas12a) and the K607R‐mutated *Acidaminococcus* sp. Cas12a (Cas12a K607R) constructs were transformed into *E. coli* T7 competent cells for protein expression. Single colonies were cultured in LB medium with ampicillin overnight at 37 °C. A 1 mL aliquot of the overnight culture was inoculated into 500 mL of 2×YT broth and grown at 37 °C until the optical density at 600 nm (OD_600_) reached ≈0.4. Protein expression was induced by the addition of 0.5 mm IPTG, followed by overnight incubation at 16 °C to facilitate soluble protein production.

Cells were harvested by centrifugation and resuspended in lysis buffer containing 50 mm Tris‐HCl (pH 7.5), 500 mm NaCl, 1 mm TCEP, 0.5 mm PMSF, 1 mg mL^−1^ lysozyme, and 5% glycerol. Cell lysis was carried out by ultrasonication on ice, and lysates were clarified by centrifugation at 16 000 ×g for 25 min at 4 °C. The supernatant was filtered through a 0.22 µm syringe filter and applied to Ni‐NTA agarose resin for His‐tag‐based affinity purification, following the manufacturer's protocol. To remove the MBP and His tags, TEV protease digestion was performed at 4 °C for 36 h. The digested protein was passed through an MBPTrap column to eliminate residual tags, followed by heparin affinity chromatography for further purification. The final product was concentrated via ultrafiltration and assessed for purity by SDS‐PAGE. Both WT Cas12a and Cas12a K607R proteins were stored in buffer containing 20% glycerol at −20 °C until use.^[^ ^42^ ^]^

### Synthesis of Metal‐Organic Frameworks (MOFs)

The organic linker (squaric sodium) was first synthesized by neutralizing squaric acid with sodium hydroxide in aqueous solution. Specifically, squaric acid (1.6 mmol, 182 mg) and NaOH (3.2 mmol, 128 mg) were dissolved in 16 mL of deionized water under room temperature conditions with constant stirring. After complete dissolution, the solution was left to evaporate at room temperature until ≈80% of the solvent volume was removed. The resulting supersaturated solution yielded fine microcrystalline squaric sodium, which was collected and stored in a desiccator until further use. This linker was subsequently employed in the synthesis of squarate‐based MOFs with various metal ions.

Squaric sodium solution (30 mg mL^−1^, 0.14 m) was prepared in Milli‐Q water. Separate 0.14 m metal precursor solutions were prepared in aqueous ethanol (10%) using the following salts: MnCl_2_·4H_2_O, MgCl_2_·6H_2_O, CaCl_2_·2H_2_O, CuCl_2_·2H_2_O, FeSO_4_·7H_2_O, Zn(NO_3_)_2_·6H_2_O. For each MOF, the squaric sodium solution (250 µL) was mixed with the respective metal salt solution (250 µL). The mixtures were vortexed briefly and allowed to react at room temperature for 30 min. An extended incubation time of 4 h was used specifically for Mg^2^⁺ to ensure complete MOF formation due to its slower coordination kinetics. After incubation, all samples were centrifuged at 10 000 rpm for 10 min, and the resulting MOFs were washed three times with Milli‐Q water. The washed products were dried under a vacuum overnight before further use.

### Encapsulation of Cas12a‐crRNA RNP in Metal Organic Frameworks (RNP@MOFs)

Cas12a protein (0.8 µm) and crRNA (1.0 µm) were preassembled in a custom buffer containing 10 mm Tris‐HCl and 50 mm NaCl (pH 7.9). The mixture was incubated at room temperature for 15 min to allow the complete formation of the Cas12a‐crRNA RNP complex. For MOF encapsulation, the preformed RNP solution (250 µL) was mixed with metal ion solution (250 µL, 0.14 m for Mn, Mg, Ca, Cu, Fe, or Zn; prepared in 10% aqueous ethanol). Immediately after, squaric sodium solution (0.14 m, 250 µL) was added under gentle vortexing. The mixture was allowed to stand at room temperature for 30 min to promote coordination‐driven MOF assembly and RNP encapsulation. For Mg^2^⁺, the incubation was extended to 4 h due to slower coordination kinetics. The resulting RNP@MOF composites were collected by centrifugation at 10 000 rpm for 10 min, followed by three washes with nuclease‐free water. The dried pellets were stored at 4 °C or resuspended in reaction buffer (0.4 mg mL^−1^) prior to downstream CRISPR fluorescence assays.

### Bacterial Culture

*Escherichia coli* (*E. coli*), *Pseudomonas aeruginosa* (*P. aeruginosa*), *Listeria monocytogenes* (*L. monocytogenes*), and *Bacillus subtilis* (*B. subtilis*) were streaked on LB agar plates and incubated overnight at 37 °C. A single colony from each strain was inoculated into LB broth and cultured at 37 °C with shaking until the cells reached the mid‐logarithmic phase. Bacterial concentrations were estimated by measuring optical density at 600 nm (OD600), where an OD600 of 0.2 was approximated to 10^7^ CFU mL^−1^. Harvested cells were subsequently used for DNA extraction, bacterial lysis, and CRISPR‐based detection assays.

### Characterization of the Cis‐ and Trans‐Cleavage Activity

For the *cis*‐cleavage assays, a 371 bp double‐stranded DNA (dsDNA) amplicon was generated using universal primers targeting a conserved 16S rRNA region upstream of the Shine‐Dalgarno sequence. The PCR product was purified using the DNA Clean‐Up Kit (New England Biolabs). The cleavage reaction was performed by combining WT Cas12a or Cas12a K607R (100 nm), crRNA (100 nm), and dsDNA target (10 nm) in 1× NEBuffer 2.1 in a total volume of 20 µL. The reaction was incubated at 37 °C for 30 min. Samples were resolved on a 2% agarose gel and stained with Gel Red. Successful cleavage was indicated by two distinct bands at 236 and 135 bp. For the *trans*‐cleavage analysis, Cas12a:crRNA RNP complexes were assembled by incubating 100 nm Cas12a with 100 nM crRNA in 1× NEBuffer 2.1 at room temperature for 10 min. A circular single‐stranded DNA substrate (M13mp18, NEB) was added at 10 nm and incubated at 37 °C for up to 60 min. Reactions were stopped at indicated time points, quenched with gel loading dye, and analyzed on a 1% agarose gel to visualize degradation.

### Fluorescence Detection Assay and Optimization

To assess the *trans*‐cleavage activity, fluorescence‐based detection assays were performed in a 50 µL reaction volume. First, 20 µL of 0.4 mg mL^−1^ RNP@MOF was disassembled in 100 mm MES buffer (pH 6.0) by incubation at room temperature for 10 min. To neutralize the acidic pH and restore Cas12a enzymatic activity, 30 µL of a custom NEBuffer (pH ≈8.3) was added, yielding a final reaction pH of ≈7.9. The complete reaction mixture contained 0.4 mg mL^−1^ RNP@MOF, 60 nm single‐stranded DNA fluorophore‐quencher (ssDNA‐FQ, 5′‐FAM‐TTATT‐BHQ1‐3′) reporter, and target DNA (concentration varied based on the application). For fluorescence‐based *trans*‐cleavage activity, reactions were incubated at 37 °C and fluorescence was recorded every 2 min for 120 min using a BioTek Synergy H5 microplate reader (excitation: 485 nm, emission: 528 nm). Optimized reactions were incubated at 40 °C for 120 min, and fluorescence was measured at the endpoint using the reader. All measurements were performed using three independent biological replicates (*n* = 3), unless otherwise specified. All fluorescence values were normalized against the no‐target control (F/F_0_), and experiments were conducted in triplicate unless otherwise stated. Endpoint tube images were also captured to visualize fluorescence output.

### Stability Evaluation of MOF‐Encapsulated CRISPR‐Cas12a

The stability of the Mn‐MOF–encapsulated Cas12a K607R:crRNA ribonucleoprotein (CRISPR‐FLEXMO) was assessed under heat, solvent, enzymatic, chaotropic, and storage stress conditions. Unencapsulated RNP served as the control. To assess thermal stability, MOF@RNP and free RNP (100 nm Cas12a, 60 nm crRNA) were incubated at 25, 40, 50, 60, 70, or 80 °C for 30 min. Following treatment, MOF@RNP was disassembled using MES buffer (pH 6.0) for and the released enzyme was analyzed for *trans*‐cleavage activity using the fluorescence assay described in Section. Solvent Tolerance was tested by incubating MOF@RNP pellets were incubated in 300 µL of various solvents (methanol, ethanol, acetone, DMSO, and isopropanol) for 1 h. Particles were then centrifuged (10 000 × g, 10 min), washed thrice with nuclease‐free water, and resuspended in MES buffer (pH 6.0) prior to assay. Under enzymatic and chaotropic stress, MOF@RNP complexes were exposed to 4 mg mL^−1^ of Proteinase K, RNase A, or trypsin, and to chaotropic agents (6 m urea, 6 m guanidinium chloride, 1% SDS) for 1 h. MOF@RNP was washed and released in MES buffer before fluorescence analysis. Free RNP was also followed by similar treatment. To evaluate long‐term storage stability, MOF@RNP complexes were stored in dry form at ambient temperature (≈25 °C) for up to 12 weeks. At weekly intervals, samples were rehydrated in MES buffer (pH 6.0) and tested for *trans*‐cleavage activity. Unencapsulated RNPs stored in NEBuffer 2.1 at room temperature served as controls. Activity was measured using the fluorescence assay (Ex: 485 nm; Em: 528 nm) and expressed relative to freshly prepared MOF@RNP.

### Recombinase Polymerase Amplification (RPA)

RPA was performed using the TwistAmp Basic kit (TwistDx, UK) following the manufacturer's protocol with slight modifications. A total reaction volume of 47.5 µL was prepared by combining primer mix (8 µL, containing both 10 µm forward and reverse primers), rehydration buffer (24.5 µL), processed clinical samples as DNA template (2 µL), and nuclease‐free water (9 µL). This mixture was added to a lyophilized RPA pellet supplied in the kit. To initiate the reaction, magnesium acetate (2.5 µL of 280 mm) was added, followed by brief vortexing. The mixture was incubated at 39 °C for 20 min. A brief vortexing step was included at 4 min to ensure homogeneity. For template input, supernatant (2 µL) obtained from heat‐lysed bacterial serum samples was used.

### Quantitative PCR (qPCR) for Clinical Validation

To benchmark the presence of bacterial DNA in clinical serum samples, SYBR Green‐based quantitative PCR (qPCR) was performed using the same universal primer pair targeting a conserved 16S rRNA region. DNA was extracted directly from 1 µL of serum per reaction without pre‐amplification. qPCR was carried out using the Luna Universal qPCR Master Mix (New England Biolabs) in a 20 µL total reaction volume, which included 10 µL of 2× Luna Master Mix, 0.2 µm of each primer, 1 µL of template DNA (serum), and nuclease‐free water to volume. Reactions were run on a Bio‐Rad CFX96 Real‐Time PCR Detection System under the following thermal cycling conditions: initial denaturation at 95 °C for 60 s, followed by 40 cycles of denaturation at 95 °C for 15 s and annealing/extension at 60 °C for 30 s. Melt curve analysis was performed to confirm amplification specificity. All clinical samples (*n* = 18; 15 patients and 3 healthy controls) were tested in technical duplicates. Successful amplification in patient samples and absence of signal in healthy controls confirmed the diagnostic relevance and specificity of the universal primer set.

### Clinical Sample Processing

Clinical serum specimens from sepsis patients were collected with signed informed consent and approval from the University of California, Riverside (UCR, #HS‐17‐707), and Riverside University Health System (RUHS, #1024190‐3) Institutional Review Board.^[^ ^43^ ^]^ For detection in clinical matrices, human serum (20 µL) was transferred into a 1.5 mL microcentrifuge tube and mixed with lysis buffer (1% Triton X‐100 in TE buffer, 10 µL). The mixture was heated at 95 °C for min to ensure bacterial lysis. After heating, the samples were centrifuged at 12 000 × g for 5 min, and the resulting supernatant was carefully transferred to a new tube. An aliquot (2 µL) of this supernatant was used as the template input for RPA amplification or direct CRISPR‐based detection.

### Statistical Analysis

All experiments were independently repeated at least three times unless otherwise stated. Fluorescence‐based measurements are presented as mean ± standard deviation (S.D.). For comparative analysis between two groups, unpaired Student's t‐tests were performed. ^*^ *p* < 0.05 and ^**^ *p* < 0.01. Relative activity was calculated by normalizing fluorescence intensities against untreated RNP or MOF@RNP controls. Statistical analysis and graphical plotting were performed using GraphPad Prism 8.0 (GraphPad Software, USA) and OriginPro 2025 (OriginLab Corporation, USA). ImageJ (Fiji) software was used to measure MOF particle size distributions.

## Conflict of Interest

The authors declare no conflict of interest.

## Supporting information



Supporting Information

## Data Availability

The data that support the findings of this study are available from the corresponding author upon reasonable request.
