# Supplementary material for: CRISPR in MOF Formulation with Enhanced Stability, Activity, and Altered PAM Specificity for Broad‐Spectrum Diagnosis of Bacterial Sepsis
Source: Adv Sci (Weinh). 2025 Nov 23;13(8):e13439. doi: 10.1002/advs.202513439 (PMC12884783; doi:10.1002/advs.202513439)
Supplement: Supplementary file 1 — Supporting Information [file ADVS-13-e13439-s001.docx]

**Supporting Information**

CRISPR in MOF Formulation with Enhanced Stability, Activity, and Altered PAM Specificity for Broad-Spectrum Diagnosis of Bacterial Sepsis

Tathagata Pal*^a^, Zilong Liu^a^, Meera G. Nair^b^, Juhong Chen*^a^

^a^ Department of Bioengineering, University of California Riverside, Riverside, CA 92521, USA

^b^ Division of Biomedical Sciences, School of Medicine, University of California, Riverside, Riverside, CA 92521, USA

*Corresponding author: T. Pal (prameyo@gmail.com)

*Corresponding author: J. Chen (jchen@ucr.edu)

| **Sl. No.** | **Content** | **Page No.** |
| --- | --- | --- |
| 1 | **Figure S1** Agarose gel electrophoresis confirming universal amplification of the 16S rRNA region across representative Gram-negative and Gram-positive bacterial strains using a single primer set. | S4 |
| 2 | **Figure S2.** Sequence alignment of the conserved 16S rRNA region upstream of the Shine-Dalgarno sequence from four representative bacterial species. | S5 |
| 3 | **Figure S3.** Sanger sequencing confirmation of the K607R point mutation in the *AsCas12a* gene. | S6 |
| 4 | **Figure S4.** SDS-PAGE analysis of Cas12a protein purification. (a) WT Cas12a purification (b) K607R Cas12a purification. | S7 |
| 5 | **Figure S5.** Synthesis of squaric sodium organic linker. | S8 |
| 6 | **Figure S6.** Color image of squarate-based metal-organic frameworks (MOFs) synthesized with six different metal ions before and after CRISPR ribonucleoprotein (RNP) encapsulation. | S9 |
| 7 | **Figure S7** Scanning electron microscopy (SEM) images showing morphological comparison of squarate-based MOFs synthesized using six different metal ions, with and without CRISPR-Cas12a ribonucleoprotein (RNP) encapsulation. Metal ions used: Mn, Mg, Ca, Cu, Fe, Zn | S10 |
| 8 | **Figure S8** XRD patterns of squarate-based MOFs synthesized with six metal ions. | S11 |
| 9 | **Figure S9** SEM micrographs of Mn-MOF with encapsulation for size distribution measurement | S12 |
| 10 | **Figure S10** Apparent zeta potential of encapsulated Mn MOF | S13 |
| 11 | **Figure S11.** Calibration curve for bicinchoninic acid (BCA) assay with absorbance value within 1 for calculation of encapsulation efficiency of RNP in MOF | S14 |
| 12 | **Figure S12.** Component-specific fluorescence analysis of the MOF-CRISPR assay. | S15 |
| 13 | **Figure S13.** Comparison of CRISPR *trans*-cleavage activity between wild-type Cas12a and K607R variant encapsulated in Mn-MOFs. | S16 |

| **Sl. No.** | **Content** | **Page No.** |
| --- | --- | --- |
| 14 | **Figure S14.** Effect of Mn^2+^ concentration on Cas12a-crRNA RNP activity. | S17 |
| 15 | **Figure S15.** Comparison of CRISPR assay performance using Mn-MOF and free Mn^2+^ ions. | S18 |
| 16 | **Figure S16**. Validation of CRISPR-FLEXMO assay across Gram-negative and Gram-positive bacterial pathogens. | S19 |
| 17 | **Figure S17.** XRD analysis of CRISPR-FLEXMO before and after stress treatments. | S20 |
| 18 | **Figure S18.** Assessment of assay specificity against viral nucleic acids. | S21 |
| 19 | **Table S1.** Universal Primer Properties for Broad-Spectrum Bacterial Detection from NCBI Primer-BLAST | S22 |
| 20 | **Table S2.** Sequences used in this study | S23 |
| 21 | **Table S3** Limit of detection calculation | S24 |
| 22 | **Table S4.** Comparison of protein preservation methods with the MOF-based CRISPR-FLEXMO approach. | S25 |
| 23 | **Table S5** Summary of primary clinical diagnoses associated with sepsis patient cohort. | S26 |
| 23 | **Table S6.** Comparison of CRISPR-FLEXMO, LAMP-CRISPR and rapid immunoassay platforms | S27 |
| 25 | **References** (Supplementary Information) | S28 |


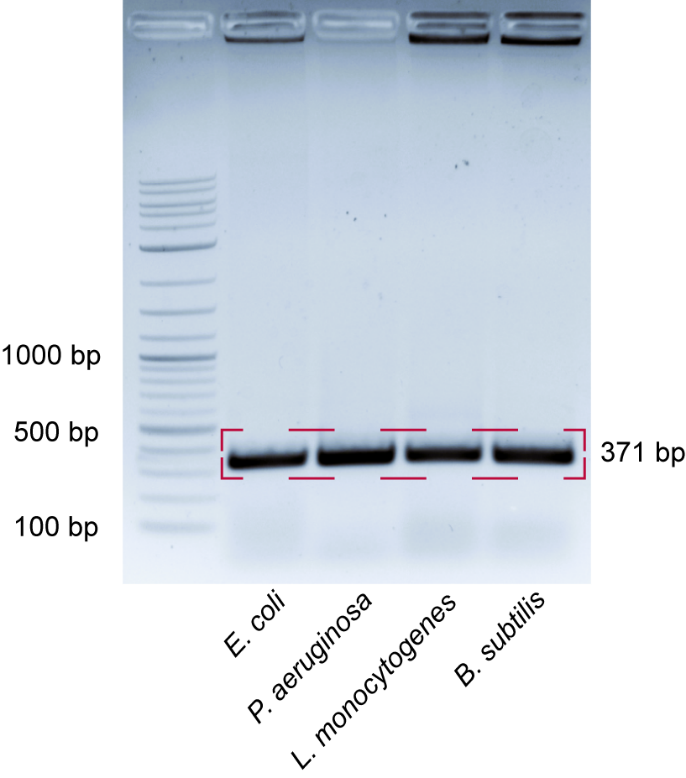


**Figure S1.** Agarose gel electrophoresis confirming universal amplification of the 16S rRNA region across representative Gram-negative (*Escherichia coli* and *Pseudomonas aeruginosa*) and Gram-positive (*Listeria monocytogenes* and *Bacillus subtilis*) bacteria using a single primer set. All lanes show a distinct band with a size of 371 bp, validating the broad-spectrum compatibility of the designed universal primers.


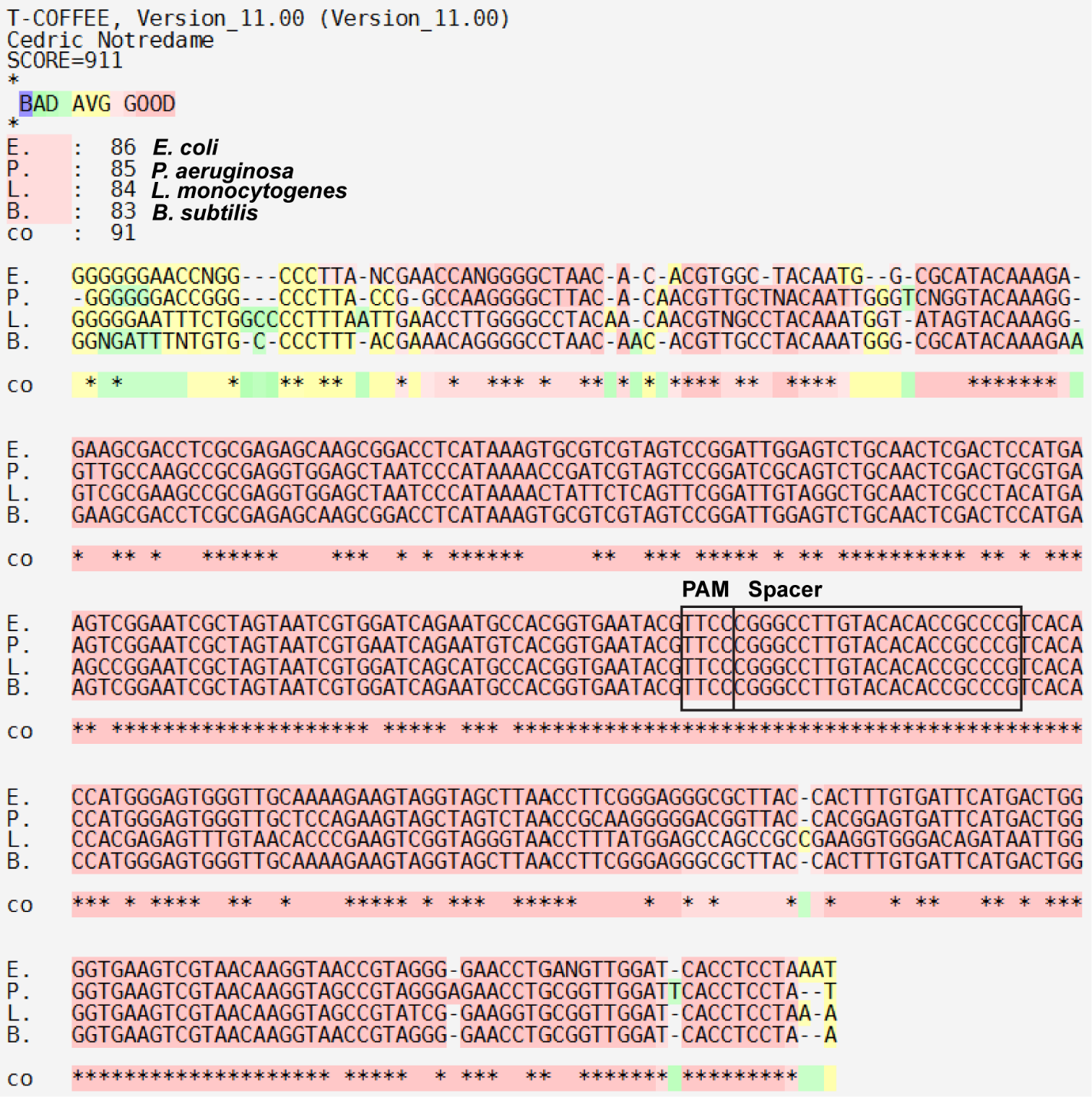


**Figure S2.** Sequence alignment of the conserved 16S rRNA region upstream of the Shine-Dalgarno sequence from four representative bacterial species. The alignment was generated using T-Coffee (Tree-based Consistency Objective Function for alignment Evaluation), version 11.00. Sequencing was performed using the reverse primer on gel-purified amplicon of *Escherichia coli* (E.), *Pseudomonas aeruginosa* (P.), *Listeria monocytogenes* (L.), and *Bacillus subtilis* (B.). The TTCC PAM motif and adjacent spacer region, selected for CRISPR targeting, are highlighted. The high conservation across both Gram-negative and Gram-positive bacteria supports the universality of the primer pair and CRISPR target site used in this study.


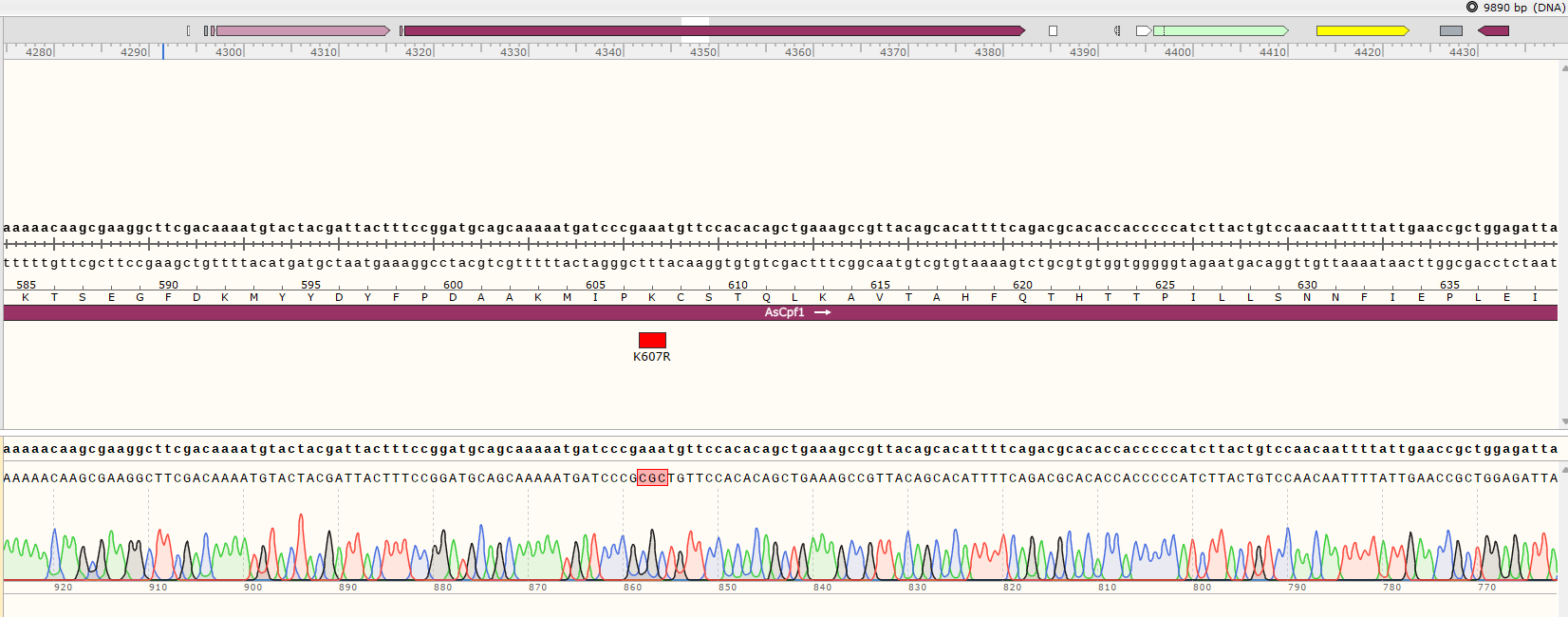


**Figure S3.** Sanger sequencing confirmation of the K607R point mutation in the *AsCas12a* gene. The sequencing trace and aligned sequence show the successful replacement of the wild-type AAA codon (lysine, K) with CGC (arginine, R) at position 607. The mutated codon is highlighted and annotated as “K607R” within the *AsCas12a* open reading frame. Alignment and visualization were performed using SnapGene® software.

**
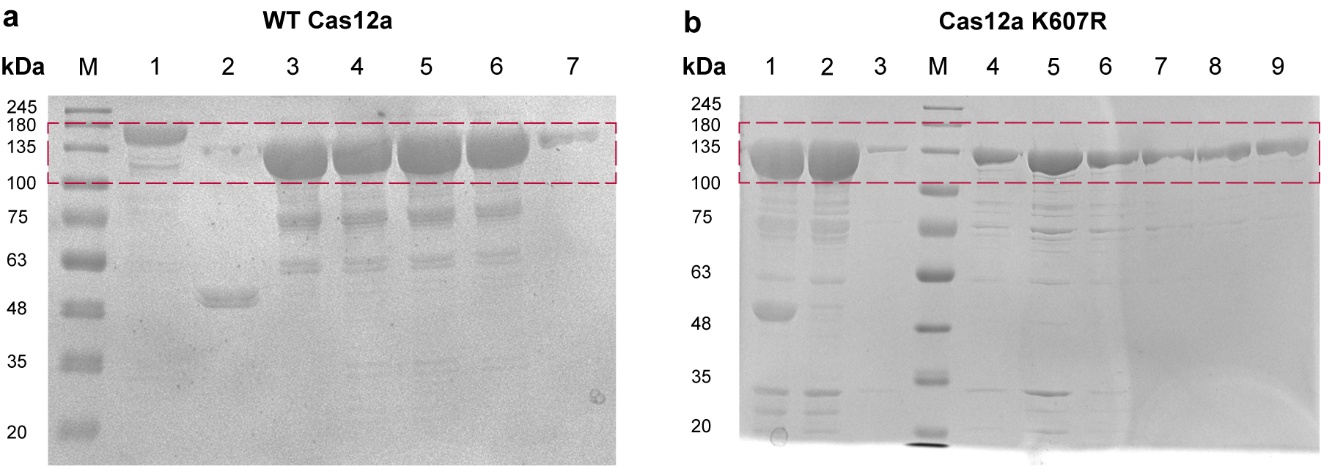
**

**Figure S4.** SDS-PAGE analysis of Cas12a protein purification. (a) WT Cas12a purification steps. Lanes 1-2: post-TEV protease cleavage and MBPTrap-purified fractions, respectively. Lanes 3-6: elution fractions from MBPTrap column. Lane 7: final concentrated protein sample. Lane M: protein molecular weight marker. (b) Cas12a K607R purification steps. Lane 1: post-TEV protease cleavage. Lane 2: flow-through after MBPTrap, prior to HeparinTrap purification. Lane 3: HeparinTrap start buffer. Lanes 4-9: eluted protein fractions from HeparinTrap column. Lane M: protein molecular weight marker. Red dashed boxes highlight the ~135 kDa band corresponding to full-length Cas12a proteins. All purified proteins were stored in 20% glycerol at -20 °C for downstream applications.


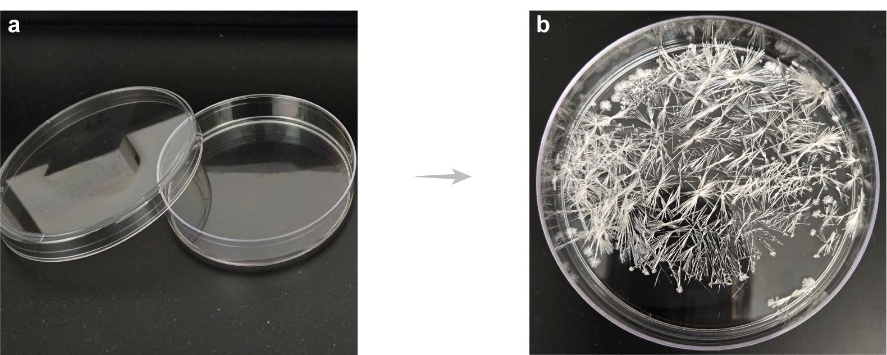


**Figure S5.** Synthesis of sodium squaric organic linker. (a) Aqueous reaction mixture containing squaric acid and sodium hydroxide in a 1:2 molar ratio, prior to evaporation. (b) Crystalline squaric sodium linker formed upon ~80% water evaporation at room temperature. The resulting microcrystals served as the organic linker for subsequent metal-organic framework (MOF) synthesis.


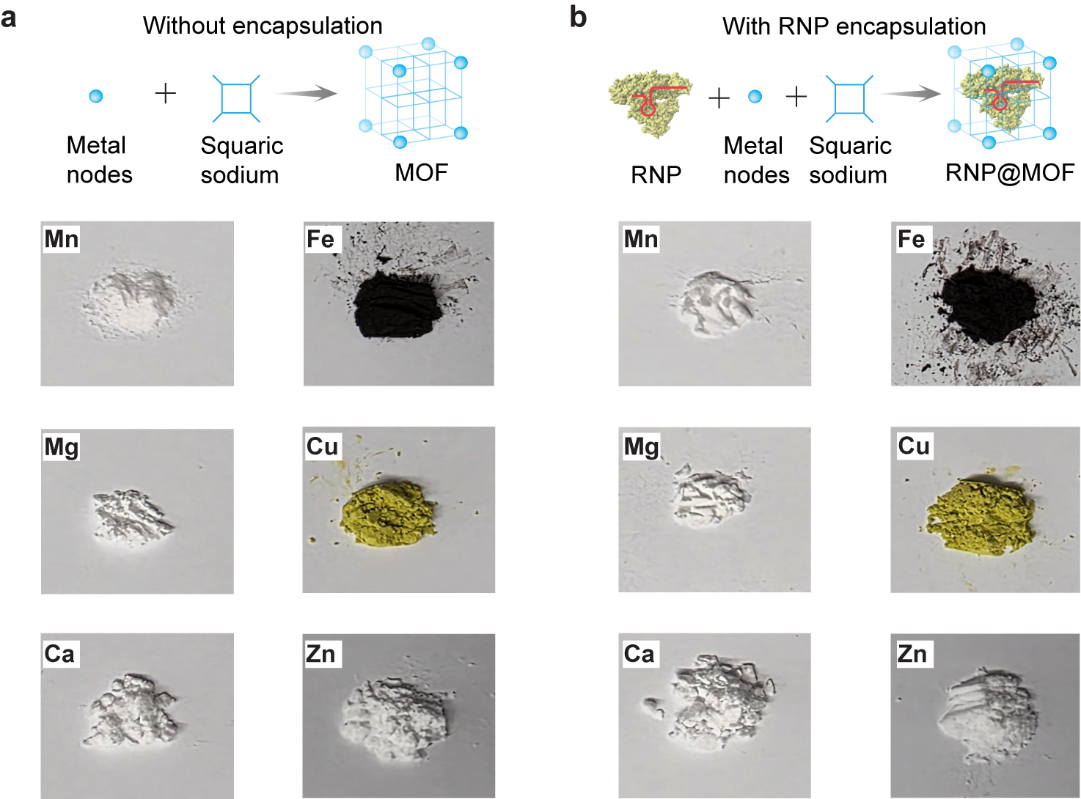


**Figure S6.** Photographic images showing the macroscopic appearance of squarate-based metal organic frameworks (MOFs) synthesized using six different metal ions, without (panel a) and with (panel b) CRISPR-Cas12a ribonucleoprotein (RNP) encapsulation. (a) MOFs formed without RNP display distinct colors depending on the metal ion: Mn, Mg, Ca, and Zn yield white powders; Fe results in a deep black precipitate; and Cu generates a characteristic yellow product. (b) Corresponding RNP@MOFs synthesized in the presence of RNP maintain similar color profiles, confirming successful encapsulation without compromising MOF integrity. The Cu-MOF retains its yellow hue, and Fe-MOF remains dark, suggesting metal-dependent optical properties. Other MOFs exhibit minimal color change, maintaining their white appearance.
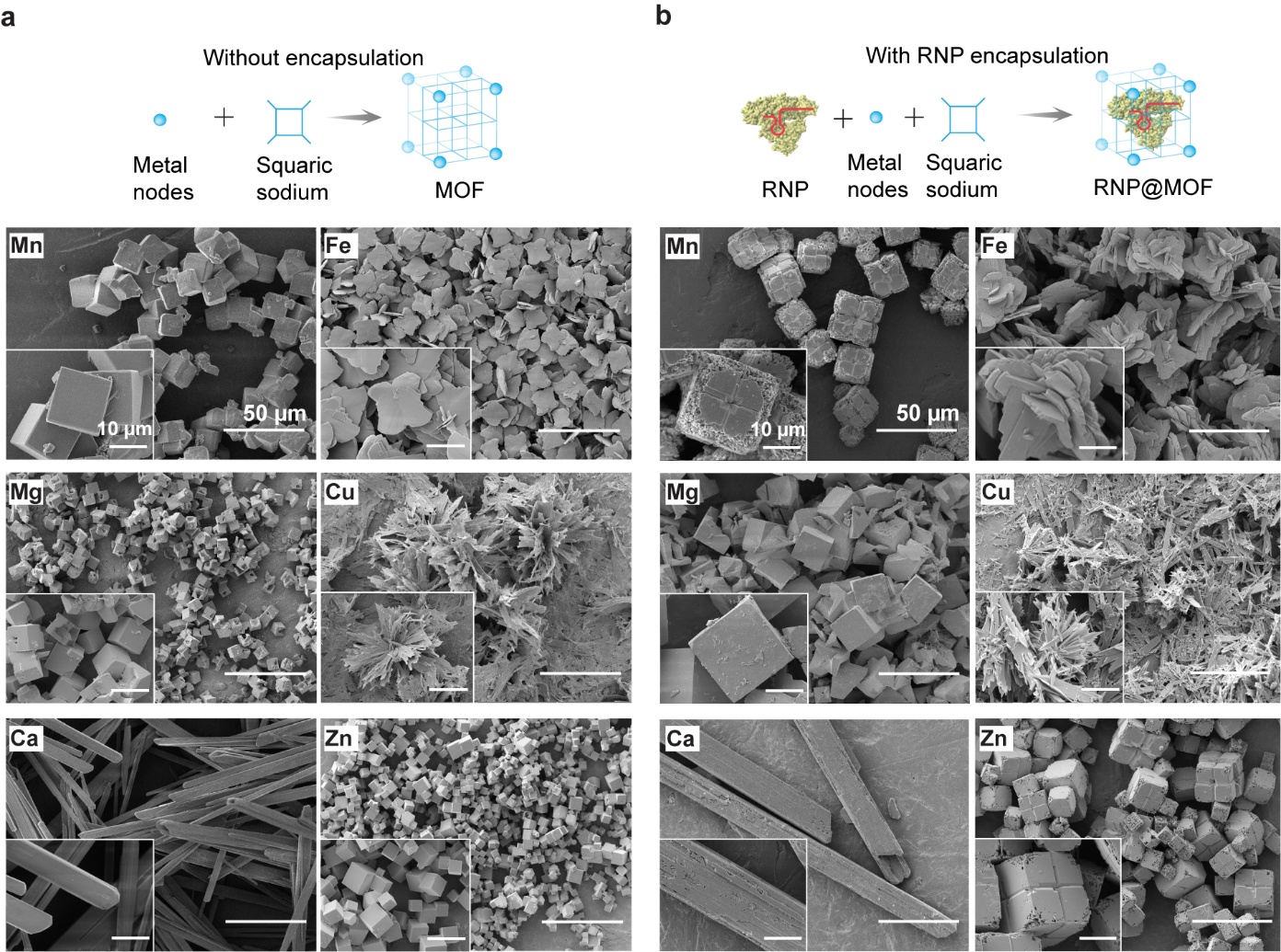


**Figure S7.** Scanning electron microscopy (SEM) images showing the morphological comparison of squarate-based metal organic frameworks (MOFs) synthesized using six different metal ions, without (panel a) and with (panel b) CRISPR-Cas12a ribonucleoprotein (RNP) encapsulation. Metal ions used: Mn, Mg, Ca, Fe, Cu, and Zn. Insets show higher magnification images highlighting crystal features. Scale bar: 50 µM for lower magnification, 10 µM for lower magnification (inset). RNP encapsulation induces surface and morphological changes such as roughening, fusion, and network restructuring, varying by metal ion. These modifications indicate successful biomolecule incorporation while preserving the underlying MOF crystallinity.


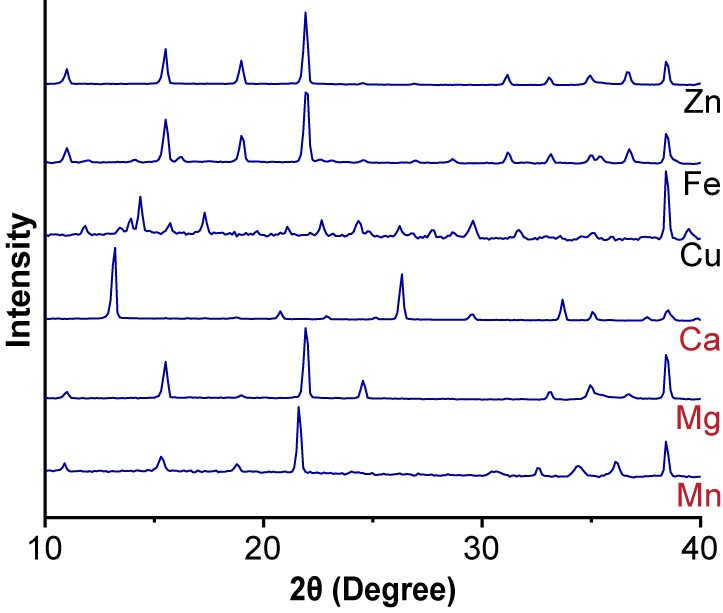


**Figure S8.** XRD patterns of squarate-based MOFs synthesized with six metal ions (without RNP encapsulation). Powder X-ray diffraction (PXRD) profiles of MOFs synthesized with Mn, Mg, Ca, Cu, Fe, and Zn confirm successful framework formation with distinct crystallinity for each composition. Similar peak positions to RNP@MOFs indicate that Cas12a/crRNA loading did not disrupt the overall framework structure.


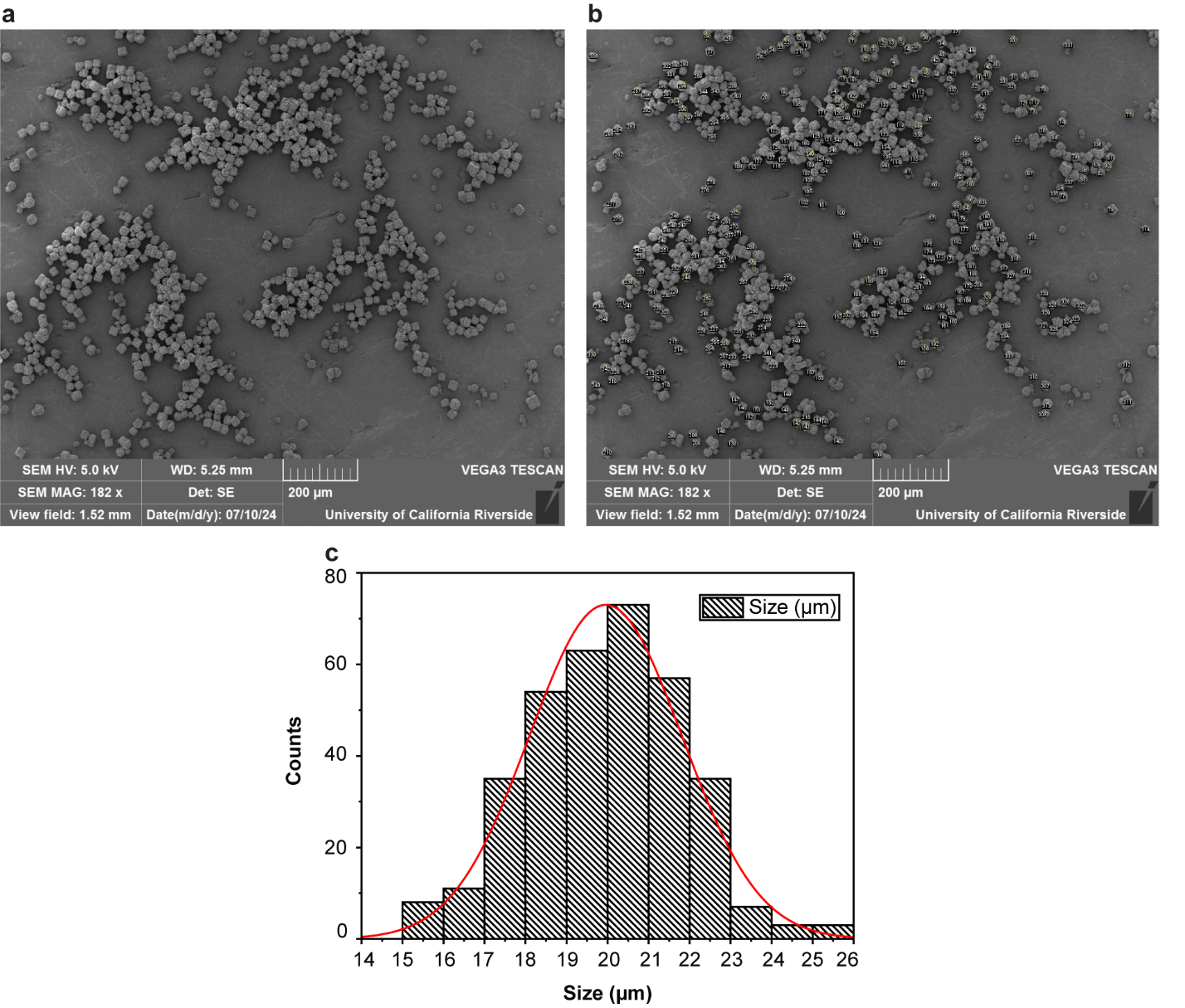


**Figure S9** Particle size distribution of Mn-MOF encapsulating the Cas12a-crRNA RNP. (a) SEM micrograph showing microcrystalline morphology of Mn-MOF. (b) SEM image with particle labelling used for image-based size analysis performed using ImageJ (Fiji) software. (c) Size distribution obtained from 349 measured particles, fitted with a Gaussian curve, showing an average particle diameter of 19.9 ± 1.9 µm (mean ± SD).


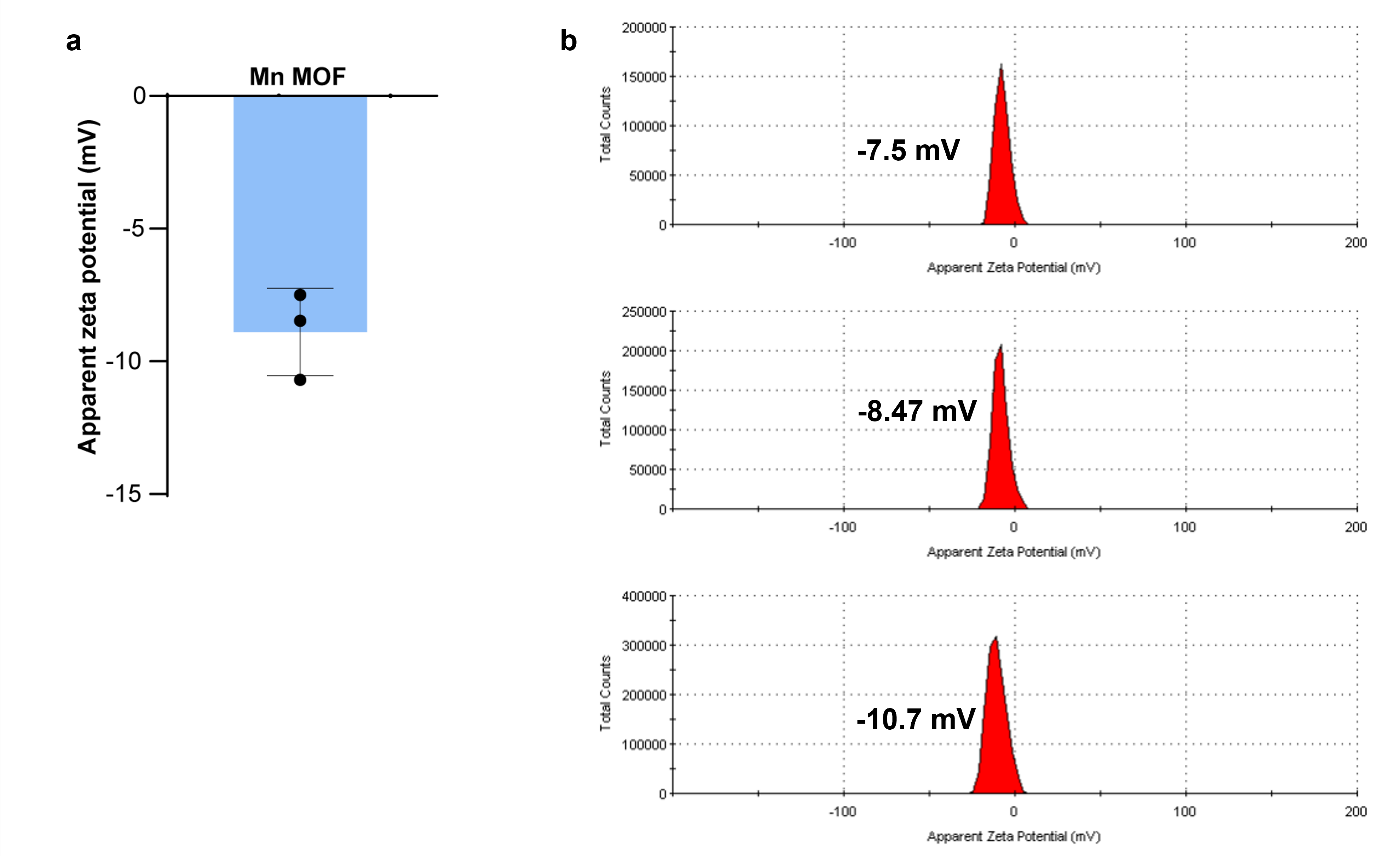


**Figure S10.** Apparent zeta potential of Mn MOF encapsulating Cas12a-crRNA RNP. (a) Mean apparent zeta potential (-8.9 ± 1.6 mV, n = 3). (b) Representative distribution plots from three independent measurements showing peaks at -7.5, -8.47, and -10.7 mV, indicating a moderately negative surface charge consistent with squarate coordination.


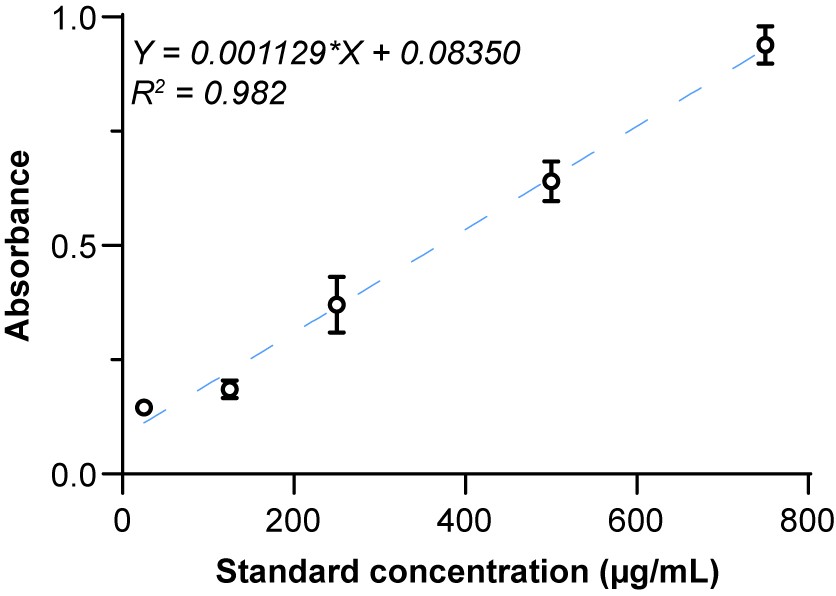


**Figure S11.** Calibration curve for bicinchoninic acid (BCA) assay with absorbance value within 1 for calculation of encapsulation efficiency of RNP in MOF.

The encapsulation efficiency of Cas12a-crRNA ribonucleoprotein (RNP) complexes within the Mn-squarate metal organic framework (Mn-MOF) was determined using the Pierce™ BCA Protein Assay. A total of 42.0 µg of RNP (0.28 nmol) was used for encapsulation under the optimized synthesis conditions, where preassembled RNP (350 µL, 0.8 µM) was mixed with 0.14 M Mn^2+^ (350 µL) and 0.14 M squaric sodium (350 µL), followed by incubation for 30 min at room temperature.

After centrifugation (6500 g, 10 min), the clear supernatant was analyzed to quantify unencapsulated RNP using a matrix-matched BCA calibration (*Y = 0.001129*X + 0.08350*). The measured absorbance corresponded to an unbound RNP mass of 4.73 µg. Subtracting this from the total input (42.0 µg) gave an encapsulated mass of 37.27 µg, resulting in an encapsulation efficiency of 88.73%.


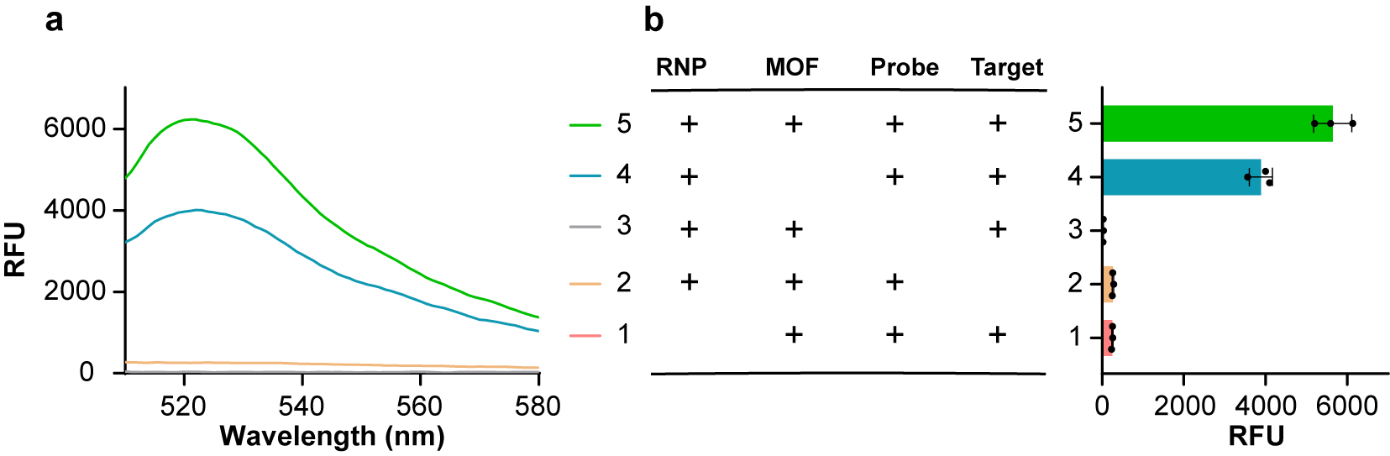


**Figure S12.** Component-specific fluorescence analysis of the MOF-CRISPR assay. (a) Fluorescence spectra of assembled assay mixtures demonstrating negligible background from MOF, probe, and RNP components in the absence of target DNA. (b) Bar graph summarizing RFU values from each condition listed in the accompanying table, confirming that significant fluorescence signal arises only in the presence of the complete CRISPR reaction (RNP, MOF, probe, and target). Error bars represent standard deviation obtained in three parallel experiments (*n* = 3).


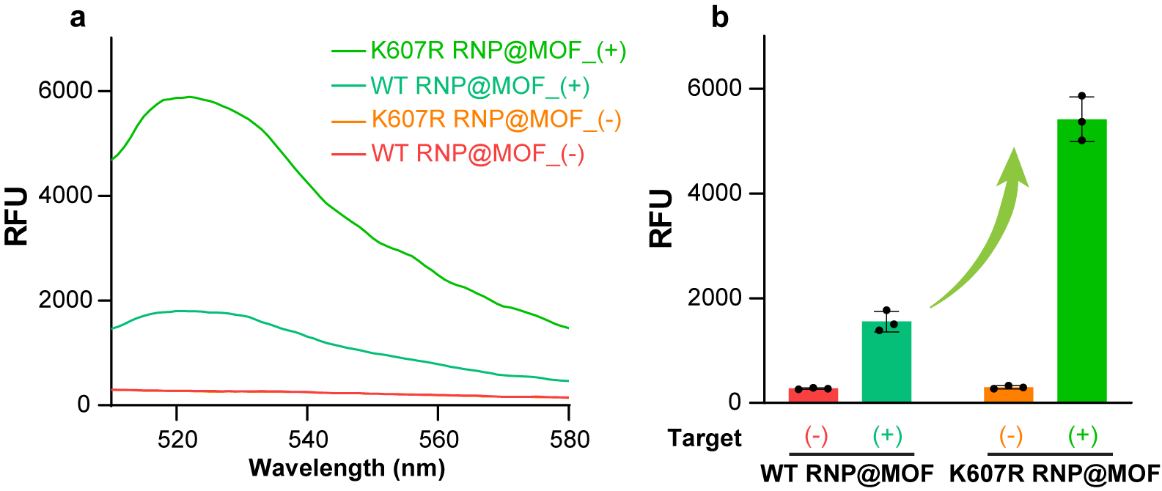


**Figure S13.** Comparison of CRISPR *trans*-cleavage activity between wild-type Cas12a and K607R variant encapsulated in Mn-MOFs. (a) Fluorescence spectra showing higher signal intensity for K607R in the presence of target DNA. (b) Endpoint fluorescence intensities confirm superior catalytic output from the engineered K607R Cas12a variant. Error bars represent standard deviation obtained in three parallel experiments (*n* = 3).


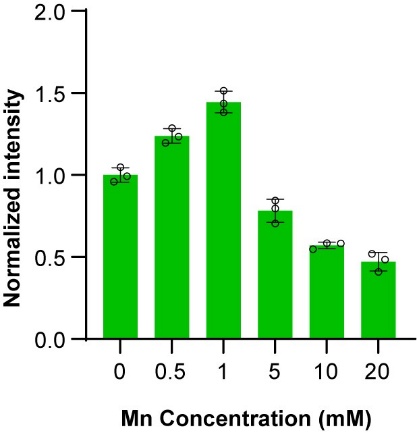


**Figure S14.** Effect of Mn^2+^ concentration on Cas12a-crRNA RNP activity. Normalized fluorescence intensity of the CRISPR assay at different Mn^2+^ concentrations (0-20 mM). Signal enhancement was observed up to 1 mM Mn^2+^, while higher concentrations (≥5 mM) reduced fluorescence, likely due to RNP denaturation. Error bars represent standard deviation obtained in three parallel experiments (*n* = 3).


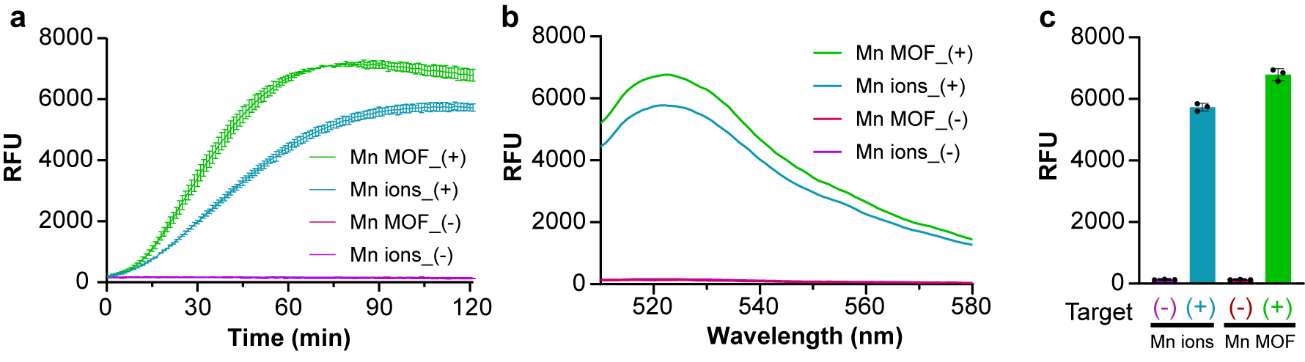


**Figure S15.** Performance comparison of the CRISPR assay using Mn-MOF and free Mn^2+^ ions. (a) Time-dependent fluorescence profiles, (b) emission spectra, and (c) endpoint RFU values showing higher signal intensity for Mn-MOF-assisted detection than for assays supplemented with 1 mM Mn^2+^. Error bars represent standard deviation obtained in three parallel experiments (*n* = 3).


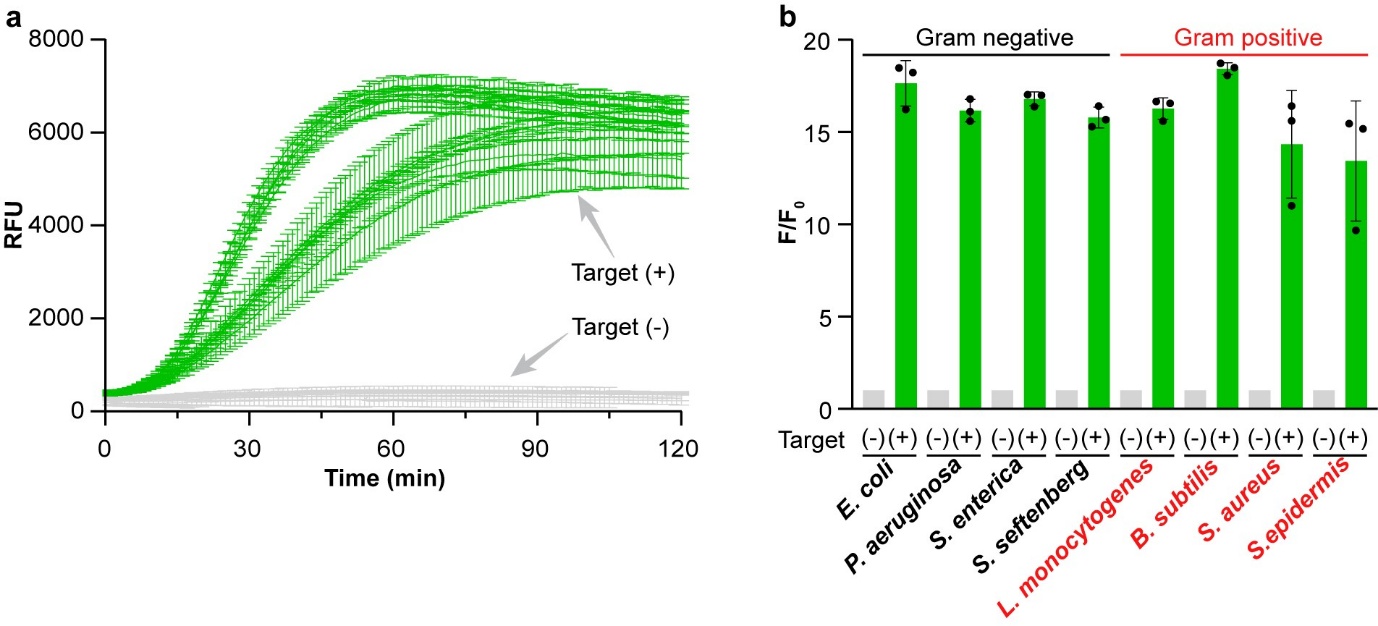


**Figure S16**. Validation of CRISPR-FLEXMO assay across Gram-negative and Gram-positive bacterial pathogens. (a) Time-resolved fluorescence curves showing robust signal generation for target-positive samples. (b) Endpoint fluorescence intensities for four Gram-negative bacteria (*Escherichia coli, Pseudomonas aeruginosa, Salmonella enterica,* and *Salmonella typhimurium*) and four Gram-positive bacteria (*Listeria monocytogenes, Bacillus subtilis, Staphylococcus aureus,* and *Staphylococcus epidermidis*), confirming broad detection capability across both bacterial classes. Error bars represent standard deviation obtained in three parallel experiments (*n* = 3).

**
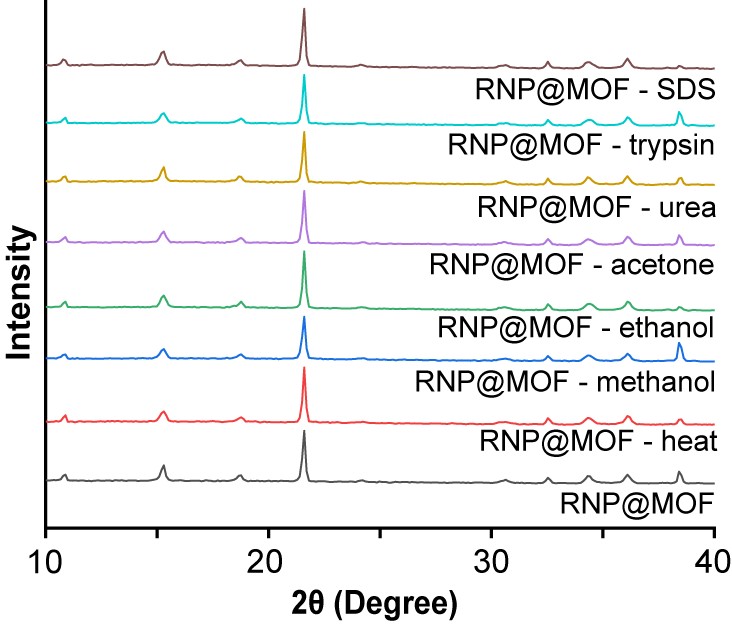
**

**Figure S17.** XRD analysis of CRISPR-FLEXMO before and after stress treatments. X-ray diffraction (XRD) patterns of RNP@Mn-squarate-MOFs following exposure to various destabilizing agents, including heat (80 °C), solvents (methanol, ethanol, acetone), and chaotropic/proteolytic agents (urea, trypsin, SDS), demonstrate retention of crystallinity. The conserved diffraction peaks confirm that the MOF structure remains intact under all tested perturbation conditions. Error bars represent standard deviation obtained in three parallel experiments (*n* = 3).


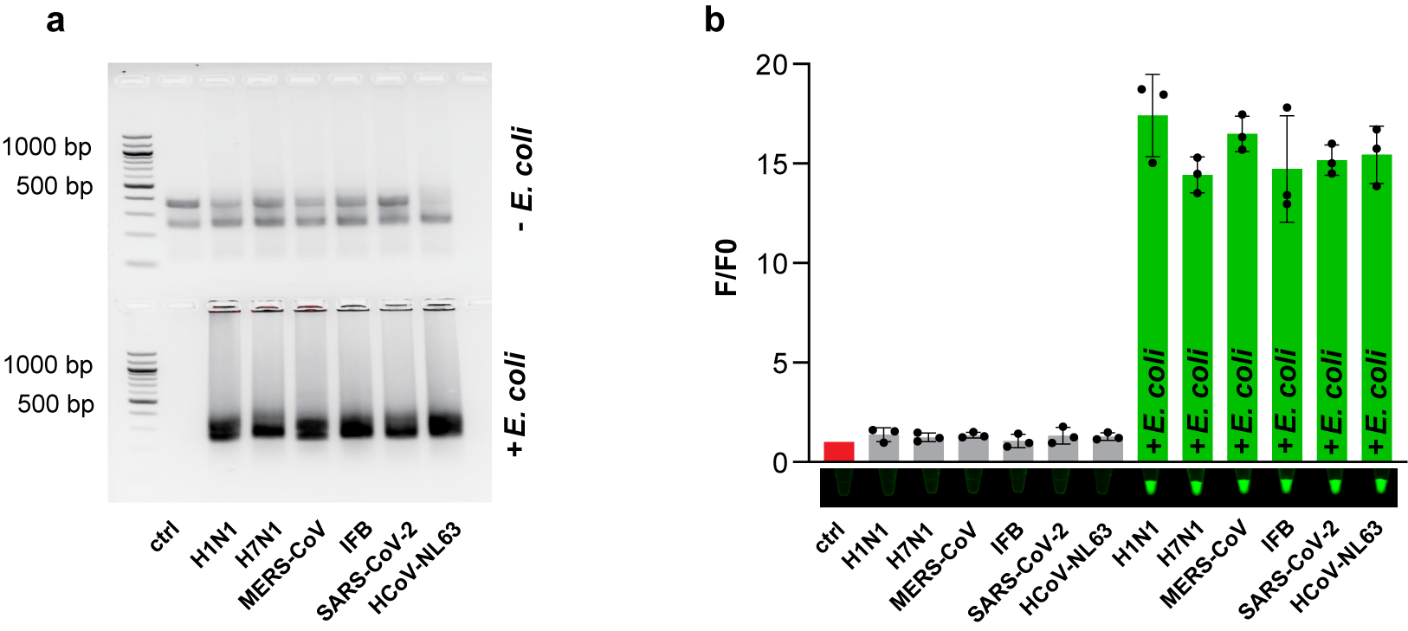


**Figure S18.** Assessment of assay specificity against viral nucleic acids. (a) RPA amplification using the universal primer set on *E. coli* and six viral targets (H1N1, H7N1, Influenza B, MERS-CoV, SARS-CoV-2, and HCoV-NL63). Non-specific primer amplicons were observed across samples, but strong bands were detected only in *E. coli*. (b) Fluorescence readout from the MOF-CRISPR assay shows selective activation in the presence of *E. coli*, with no collateral signal generated from viral templates from the non-specific bands. Inset: endpoint fluorescence image. Error bars represent standard deviation obtained in three parallel experiments (*n* = 3).

**Table S1. The properties of the universal primer for broad-spectrum bacterial detection from NCBI primer-blast**

| **Property** | **Forward Primer** | **Reverse Primer** |
| --- | --- | --- |
| Primer | Forward | Reverse |
| Sequence (5′→3′) | AACTGGAGGAAGGTGGGGA | AGGAGGTGATCCAACCGCA |
| Length (nt) | 19 | 19 |
| Melting Temperature (Tm, °C) | 59.76 | 60.92 |
| GC Content (%) | 57.89 | 57.89 |
| Self-Complementarity | 2 | 4 |
| 3′ Self Complementarity | 0 | 0 |

**Table S2. Primer, target amplicon sequence, and crRNA used in this study**

| **Name** | **Sequence (5'-3')** |
| --- | --- |
| Sepsis_F primer | AACTGGAGGAAGGTGGGGA |
| Sepsis_R primer | AGGAGGTGATCCAACCGCA |
| Target Amplicon (*E. coli*) | AACTGGAGGAAGGTGGGGATGACGTCAAGTCATCATGGCCCTTACGACCAGGGCTACACACGTGCTACAATGGCGCATACAAAGAGAAGCGACCTCGCGAGAGCAAGCGGACCTCATAAAGTGCGTCGTAGTCCGGATTGGAGTCTGCAACTCGACTCCATGAAGTCGGAATCGCTAGTAATCGTGGATCAGAATGCCACGGTGAATACGTTCCCGGGCCTTGTACACACCGCCCGTCACACCATGGGAGTGGGTTGCAAAAGAAGTAGGTAGCTTAACCTTCGGGAGGGCGCTTACCACTTTGTGATTCATGACTGGGGTGAAGTCGTAACAAGGTAACCGTAGGGGAACCTGCGGTTGGATCACCTCCT |
| crRNA | rUrUrCrCrGrGrGrCrCrUrUrGrUrArCrArCrArCrGrCrCrGr |
| ssDNA-FQ Probe | 56-FAM/TTATT/3IABkFQ |


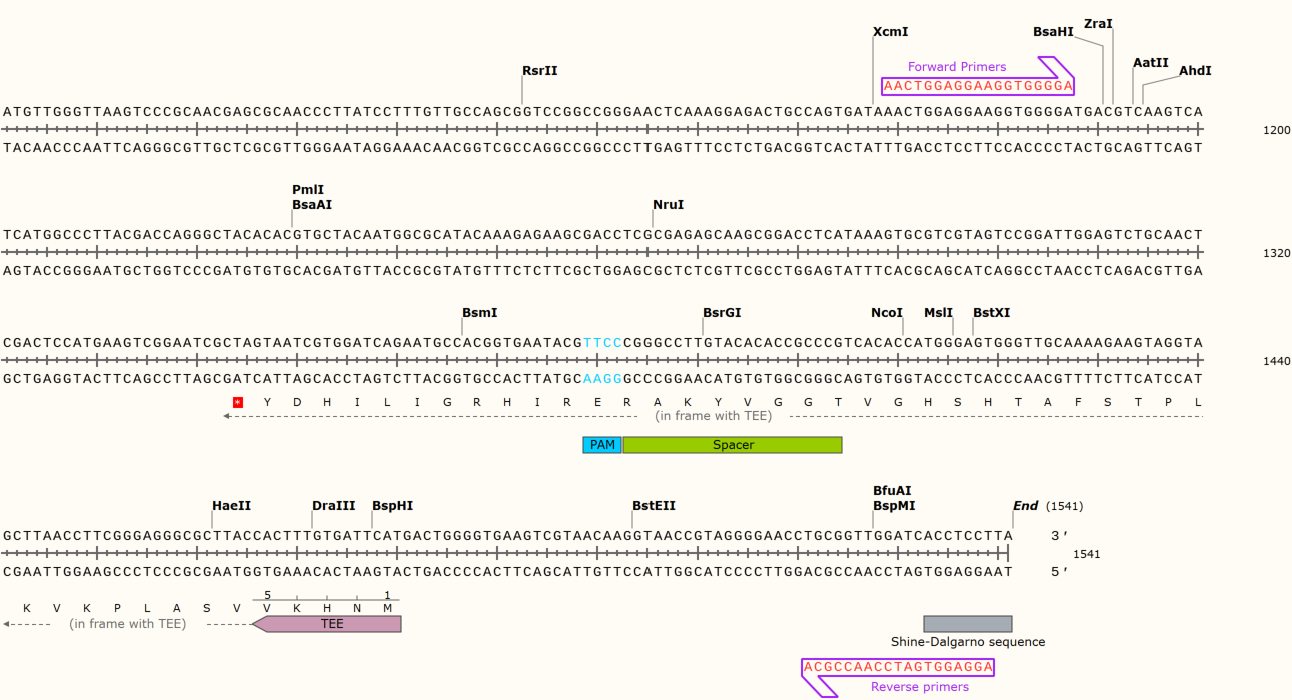
**Visualization of PAM and spacer sequences in target DNA using SnapGene software**

**Table S3. Limit of detection calculation**

| Organism | LDR (nM) | Regression Equation* | R^2^ | LOD Calculation** | LOD (nM) | *P* Value |
| --- | --- | --- | --- | --- | --- | --- |
| *E. coli* | 2-30 | *Y = 0.5154*X + 2.541* | 0.9675 | 3.3*(0.9722/0.5154) | 6.22 | <0.0001 |
| *P Aeruginosa* | 1-30 | *Y = 0.5273*X + 2.296* | 0.9707 | 3.3*(0.9464/0.5273) | 5.92 | <0.0001 |
| *L. Monocytogenes* | 2-30 | *Y = 0.4238*X + 2.072* | 0.9630 | 3.3*(0.8576/0.4238) | 6.67 | <0.0001 |
| *B. Subtilis* | 2-30 | *Y = 0.5134*X + 3.099* | 0.9689 | 3.3*(0.9477/0.5134) | 6.09 | <0.0001 |

* where Y represents the fluorescent intensity over the background and X represents the DNA

** LOD was calculated by formula 3.3(S_y_/S) where S_y_ represents the standard deviation of the response from the negative control, and S represents the slope.

**Table S4. Comparison of protein preservation methods with the MOF-based CRISPR-FLEXMO approach.**

| **Parameter** | **Freezing  (+Glycerol)** | **Lyophilization**  **(Freeze-Drying)** | **CRISPR-FLEXMO** |
| --- | --- | --- | --- |
| **Storage requirement** | -20 °C to -80 °C continuous cold chain | Ambient possible but moisture-sensitive; requires sealed desiccation | Ambient stable;  no cold chain or desiccation required |
| **Protective mechanism** | Glycerol cryoprotection against ice-crystal stress | Sugar matrix (trehalose/sucrose/BSA) replaces hydration shell | Coordination and confinement within Mn-squarate matrix prevent denaturation |
| **Freeze - thaw tolerance** | Limited; repeated cycles cause activity loss | Not applicable | High; no phase transition stress |
| **Reconstitution step** | Not needed, but must dilute glycerol before use | Required; incomplete dissolution can reduce yield | MES Buffer; pH = 6. |
| **Assay compatibility** | Glycerol ≥ 5% can inhibit enzymatic reactions | Residual excipients may affect optical assays | No inhibitory components; directly assay-compatible |
| **Stability under field conditions** | Loses activity upon accidental thawing | Degrades under humidity or heat without packaging | Stable under moderate humidity and heat |
| **Equipment and process cost** | Low, but requires freezers | High; lyophilizer and vacuum packaging needed | Low; simple centrifugation and mixing - no specialized hardware |
| **Reagent shelf life (typical)** | Weeks-months (frozen) | Months at 25 °C if vacuum sealed | Months at ambient temperature |
| **Suitability for POC use** | Poor (cold chain needed) | Moderate (requires careful handling) | Excellent; one-pot use ambient stable, low cost |
| **References** | Reference 1, 2 | Reference 3, 4 | This work |

**Table S5. Summary of primary clinical diagnoses associated with the sepsis patient cohort**

Detailed clinical diagnoses of the fifteen sepsis patients (P1-P15) enrolled for serum-based diagnostic validation. The cases reflect a diverse spectrum of sepsis-associated conditions, including gastrointestinal, urinary, respiratory, and cardiovascular origins, providing a representative sample of polymicrobial infection contexts relevant to broad-spectrum diagnostic evaluation.

| **Patients no.** | **Primary etiology of sepsis** |
| --- | --- |
| **P1** | Necrotizing pancreatitis |
| **P2** | Urinary Tract Infection |
| **P3** | Cardiac arrest (/septic shock) on arrival to ED |
| **P4** | Urinary Tract Infection |
| **P5** | Combined systolic and diastolic congestive heart failure with decompensation |
| **P6** | Small bowel obstruction (/septic shock) |
| **P7** | Gallstone pancreatitis |
| **P8** | Bilateral ruptured tubo-ovarian abscesses |
| **P9** | Community-acquired pneumonia |
| **P10** | Obstructive uropathy from ureteral stone |
| **P11** | Takotsubo cardiomyopathy |
| **P12** | Pyelonephritis |
| **P13** | G-tube dislodgement |
| **P14** | Infective myositis of right shoulder |
| **P15** | Acute hypoxic respiratory failure |

**Table S6. Comparison of CRISPR-FLEXMO, LAMP-CRISPR, and rapid immunoassay platforms**

| **Platform** | **Sensitivity** | **Clinical specificity** | **Turn-**  **around time** | **Per-test materials cost** | **Notes** | **References** |
| --- | --- | --- | --- | --- | --- | --- |
| CRISPR-FLEXMO | Very high analytical sensitivity  (100% tested in patient serum sample) | High; crRNA-defined  (100% tested in patient serum sample) | ~ 60 - 120 min (typical CRISPRdx workflows) | Low | Ambient-stable RNP@MOF (this work);  Broad-spectrum diagnosis is feasible. | This work |
| LAMP-CRISPR | High; LAMP boosts sensitivity but is primer-dependent | High; occasional off-target artifacts from amplification | ~ 60 min (typical CRISPRdx workflows) | Low | Needs constant-temperature heater; Broad-spectrum diagnosis is inconvenient. | Reference 5, 6 |
| Rapid Immunoassay  (lateral flow) | Moderate; varies by antigen/antibody | Variable; matrix- and timing-dependent | ~ 20 min | Very Low | Fastest but least sensitive; Broad-spectrum diagnosis is inconvenient. | Reference 7, 8 |

**REFERENCES:**

(1) Wang, M.; Sheng, Y.; Cui, H.; Li, A.; Li, X.; Huang, H. The Role of Glycerol in Preserving Proteins Needs to Be Reconsidered. *ACS Sustainable Chemistry & Engineering* **2022**, *10* (46). DOI: 10.1021/acssuschemeng.2c04695.

(2) Vagenende, V.; Yap, M. G. S.; Trout, B. L. Mechanisms of Protein Stabilization and Prevention of Protein Aggregation by Glycerol. **2009**. DOI: 10.1021/bi900649.

(3) Roy, I.; Gupta, M. N. Freeze‐drying of proteins: some emerging concerns. *Biotechnology and Applied Biochemistry* **2004**, *39* (2). DOI: 10.1042/BA20030133.

(4) Arsiccio, A.; Giorsello, P.; Marenco, L.; Pisano, R. Considerations on Protein Stability During Freezing and Its Impact on the Freeze-Drying Cycle: A Design Space Approach. *Journal of Pharmaceutical Sciences* **2020**, *109* (1). DOI: 10.1016/j.xphs.2019.10.022.

(5) Zhang, M.; Wang, H.; Wang, H.; Wang, F.; Li, Z. CRISPR/Cas12a-Assisted Ligation-Initiated Loop-Mediated Isothermal Amplification (CAL-LAMP) for Highly Specific Detection of microRNAs. *Analytical Chemistry* **2021**, *93* (22). DOI: 10.1021/acs.analchem.1c00686.

(6) Lee, S.-Y.; Oh, S.-W. Filtration-based LAMP-CRISPR/Cas12a system for the rapid, sensitive and visualized detection of Escherichia coli O157:H7. *Talanta* **2022**, *241*. DOI: 10.1016/j.talanta.2021.123186.

(7) Sohrabi, H.; Majidi, M. R.; Fakhraei, M.; Jahanban-Esfahlan, A.; Hejazi, M.; Oroojalian, F.; Baradaran, B.; Tohidast, M.; Guardia, M. d. l.; Mokhtarzadeh, A. Lateral flow assays (LFA) for detection of pathogenic bacteria: A small point-of-care platform for diagnosis of human infectious diseases. *Talanta* **2022**, *243*. DOI: 10.1016/j.talanta.2022.123330.

(8) Cao, X. E.; Ongagna-Yhombi, S. Y.; Wang, R.; Ren, Y.; Srinivasan, B.; Hayden, J. A.; Zhao, Z.; Erickson, D.; Mehta, S. A diagnostic platform for rapid, simultaneous quantification of procalcitonin and C-reactive protein in human serum. *EBioMedicine* **2022**, *76*. DOI: 10.1016/j.ebiom.2022.103867.
